# SSBlazer: a genome-wide nucleotide-resolution model for predicting single-strand break sites

**DOI:** 10.1186/s13059-024-03179-w

**Published:** 2024-02-12

**Authors:** Sheng Xu, Junkang Wei, Siqi Sun, Jizhou Zhang, Ting-Fung Chan, Yu Li

**Affiliations:** 1 Department of Computer Science and Engineering, The Chinese University of Hong Kong, 100871 Hong Kong SAR, China; 2 The CUHK Shenzhen Research Institute, Hi-Tech Park, Nanshan, 518057 Shenzhen, China; 3 Research Institute of Intelligent Complex Systems, Fudan University, 220 Handan Rd, Shanghai, 200437 China; 4 Shanghai AI Lab, 422 Jingan Rd, 200041 Shanghai, China; 5 School of Life Sciences, The Chinese University of Hong Kong, 100871 Hong Kong SAR, China; 6 State Key Laboratory of Agrobiotechnology, The Chinese University of Hong Kong, 100871 Hong Kong SAR, China; 7 Department of Computational Medicine and Bioinformatics, University of Michigan, Ann Arbor, USA

**Keywords:** Single-strand break, Genome instability, Nucleotide-resolution model

## Abstract

Single-strand breaks are the major DNA damage in the genome and serve a crucial role in various biological processes. To reveal the significance of single-strand breaks, multiple sequencing-based single-strand break detection methods have been developed, which are costly and unfeasible for large-scale analysis. Hence, we propose SSBlazer, an explainable and scalable deep learning framework for single-strand break site prediction at the nucleotide level. SSBlazer is a lightweight model with robust generalization capabilities across various species and is capable of numerous unexplored SSB-related applications.

**Supplementary Information:**

The online version contains supplementary material available at 10.1186/s13059-024-03179-w.

## Background

Single-strand breaks (SSBs) constitute the most common form of DNA damage within the genome [1]. The accumulation of SSBs caused by overstimulation of endogenous nucleases and defects in the DNA repair system would lead to genome instability, which has been implicated in multiple diseases such as cancer and neurological disorders [2]. A number of studies [3, 4] have demonstrated that SSB sites in the genome are not randomly distributed, but rather, they are enriched in regulatory elements and exon regions and vary among different cellular states. Endogenous SSBs occur during conventional activities such as DNA replication, recombination, and repair, with the primary source being oxidative attacks by endogenous reactive oxygen species (ROS) [5]. Furthermore, exogenous SSBs are the most frequent outcome of exposure to DNA-damaging agents such as ultraviolet (UV) and topoisomerase poison. In other words, SSBs can arise by the disintegration of the oxidized sugar directly or indirectly during DNA base-excision repair (BER) of oxidized bases, abasic sites, and damaged bases in various pathways [6].

If not promptly or adequately repaired, SSBs can influence cell fate through several pathways. For instance, studies have reported that SSBs can obstruct transcription and trigger cell death by inhibiting the progression of RNA polymerase [7]. Tyrosyl-DNA phosphodiesterase 1 (TDP1) and aprataxin (APTX) are integral to the SSB repair system. Numerous studies [8, 9] have disclosed that the absence of these essential molecules is apparent in various forms of cancer and neurodegenerative diseases. Despite the well-established significance of SSBs in cellular processes and human diseases, the specific cellular pathways that oversee each step of SSB restoration, including both SSB detection and repair procedures, have yet to be fully elucidated. Typically, defects in these pathways can lead to genotoxic stress, embryonic lethality, and various neurodegenerative diseases. Therefore, mapping the SSB landscape could offer fresh insights into these disease mechanisms and lay the groundwork for corresponding therapeutic approaches [10].

A number of studies [11–13] have primarily focused on double-strand breaks (DSBs) to elucidate the DNA lesion repair system. Notably, the DSB-only detection approaches [14–16] have been developed for the genome-wide mapping of DSBs. In contrast, SSBs are often perceived as less hazardous to cellular survival than DSBs, particularly in proliferating cells where SSBs are frequently seen as precursors to DSBs. During the replication process, if SSBs encounter replisomes, they can hinder replication forks, potentially leading to their transformation into DSBs. This indirect conversion into DSBs can contribute to genomic toxicity and, ultimately, cell death. However, SSBs can also directly affect disease progression. For instance, Higo et al. [17] discovered that SSB-induced DNA damage is integral to the pathogenesis of pressure overload-induced heart failure. The accumulation of unrepaired SSBs can trigger the DNA damage response system and increase the expression of inflammatory cytokines via the NF-$\kappa$B in the mouse model, leading to either apoptotic cell death or cellular senescence.

Mapping DNA lesions genome-wide is crucial for understanding damage signals and the corresponding DNA repair procedures in relation to genome context and chromatin status. Recently, several high-throughput technologies of SSBs detection, including S1 END-seq [18], SSiNGLe-ILM [19], and GLOE-Seq [20], have emerged, describing the genome-wide landscape of SSBs. END-seq [16] was originally designed for DSBs detection. However, by incorporating the single-strand-specific S1 nuclease, SSBs can be converted into DSBs. Furthermore, the use of dideoxynucleosides (ddN) can temper the SSB repair process. Therefore, S1 END-seq [18] can detect SSBs efficiently in a non-strand-specific manner. SSiNGLe-ILM [19] is the first next-generation sequencing (NGS)-based approach developed for SSB mapping and versatile for any lesion that can be converted into a nick with a free 3′-OH group. Leveraging the 3′-OH lesion, SSiNGLe-ILM can capture SSB sites in a strand-specific manner. These methods can map the positions of specific types of lesions with nucleotide-level resolution and accurately quantify the SSB sites in genome regions with a particular status. For instance, these experimental methods can yield insightful genome-wide SSB landscapes of various fundamental biological processes, capturing the SSB landscapes in numerous cellular states, including health and disease states, distinct developmental stages, and responses to typical environmental stresses. In a recent study, Wu et al. [18] used S1 END-seq to reveal that enhancer regions in neurons are hotspots for DNA single-strand break repair via PARP1 and XRCC1-dependent mechanisms. These experimental approaches can successfully depict the SSB genome landscape. However, these in vivo and in vitro detection approaches are time-consuming and unfeasible for large-scale analysis. These experimental methods are based on high-throughput sequencing, indicating a high demand for advanced sequencing equipment and restricting their widespread usage. Generally, recent studies [18, 19] have released genome-wide SSB distribution maps of multiple species and cell lines. These high-quality, well-curated datasets enable the construction of an in silico framework to depict the genome-wide SSB landscape across various species, potentially leading to further scientific discoveries.

In this study, we proposed the first computational approach for genome-wide nucleotide-resolution SSB site prediction, namely SSBlazer, which is an explainable and scalable deep learning framework for genome-wide nucleotide-resolution SSB site prediction. We demonstrated that SSBlazer could accurately predict SSB sites. The construction of an imbalanced dataset simulates the realistic application scenario and sufficiently alleviates false positive predictions. In addition, SSBlazer is a lightweight model with robust cross-species generalization ability, enabling large-scale genome-wide application in diverse species. The model interpretation analysis shows that SSBlazer captures the pattern of individual CpG in genomic contexts, which is consistent with a previous study [18] and provides novel potential insights into SSB occurrence mechanisms such as the break site motif of GGC in the center region. The successful development of SSBlazer has enabled a plethora of applications related to SSBs. In a specific case study, SSBlazer was employed on a sample of 212 vertebrate genomes, yielding intriguing putative SSB genomic landscapes. These landscapes offered a captivating proposition regarding the potential correlation between the frequency of SSBs and the hierarchy of evolution. Moreover, SSBlazer represents a valuable tool for delineating disease conditions. It can illustrate the alterations in the SSB landscape attributed to mutational events, as well as characterize the properties of pathogenic single-nucleotide polymorphisms (SNPs) that induce SSB. The web server of SSBlazer is now available for the simplified application on https://proj.cse.cuhk.edu.hk/aihlab/ssblazer/ and the future version will expand the species and integrate genomic annotation features.

## Results

### An overview of SSBlazer

**Fig. 1:**
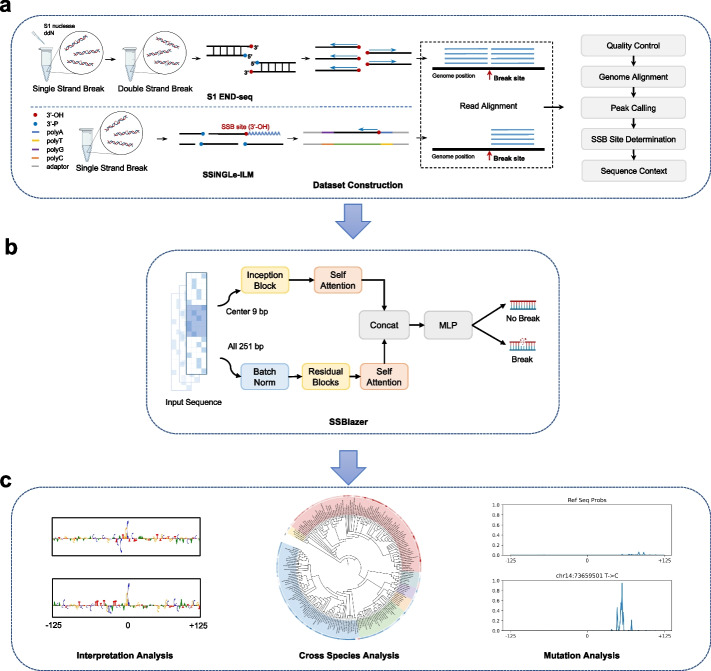
Overview of our work. **a** The experimental and bioinformatics pipeline for constructing the datasets from two different SSB detection methods (S1 END-seq and SSiNGLe-ILM). **b** The computational framework and SSBlazer. **c** The downstream analysis of the putative genome SSB landscape. For example, gradient-based model interpretation analysis reveals that the model can capture the specific motif pattern of the putative SSB sites. The cross-species analysis provided the genome-wide SSB site landscape across various genomes. The mutation analysis profiled alterations in the SSB landscape, identifying SSB-related SNPs

SSBlazer is a novel computational framework for predicting SSB sites within local genomic windows. This method utilizes advanced deep learning techniques such as residual blocks and self-attention mechanisms to enhance the accuracy of predictions. Moreover, the model is capable of quantifying the contribution of each nucleotide to the final prediction, thereby aiding in the identification of SSB-associated motifs, such as the GGC motif and regions with a high frequency of CpG sites. As illustrated in Fig. 1a, the sequencing data of S1 END-seq and SSiNGLe-ILM undergo a standard pipeline including quality control, genome alignment, peak calling, and SSB site determination to reliably capture genome-wide SSB locations [16]. To construct the sequence-based model, we extracted the sequence context of the determined SSB sites as input. During model prediction (Fig. 1b), sequences are involved in two-stream feature extraction. The full-length sequence is processed through the residual block and the self-attention block for detecting interaction within the sequence, while the center 9 bp sequence is processed through the inception block, given the central part of sequence shares higher importance, and then the self-attention block, to capture useful information from the raw sequence. Finally, these two-stream extracted feature vectors are concatenated and fed into an MLP for SSB tendency prediction. In addition, there is further interest in identifying and quantifying genomic context of SSB sites (Fig. 1c). A previous study [21] attempted to extract the motif via the activation of the first layer in the deep learning model, but this approach overlooks the complexity of the downstream layers and struggles to quantify the motif’s contribution. To address this, an activation backpropagation method (DeepLIFT [22]) was employed. The prediction of a specific sequence is backpropagated through the entire network back to the original inputs to assign contribution scores to the nucleotides. Importantly, this process considers the entire deep learning model and computes the score in a purely data-driven manner. Upon interpretation analysis, it was found that SSBlazer identifies the CpG pattern as the most crucial factor for making a positive prediction. Additionally, the model was interpolated on various vertebrate genomes, depicting the genome-wide SSB site landscapes across these genomes. Cross-species phylogenetic analysis revealed that the abundance of SSBs is indicated by the evolutionary hierarchy.

### SSBlazer accurately predicts SSB sites

**Fig. 2:**
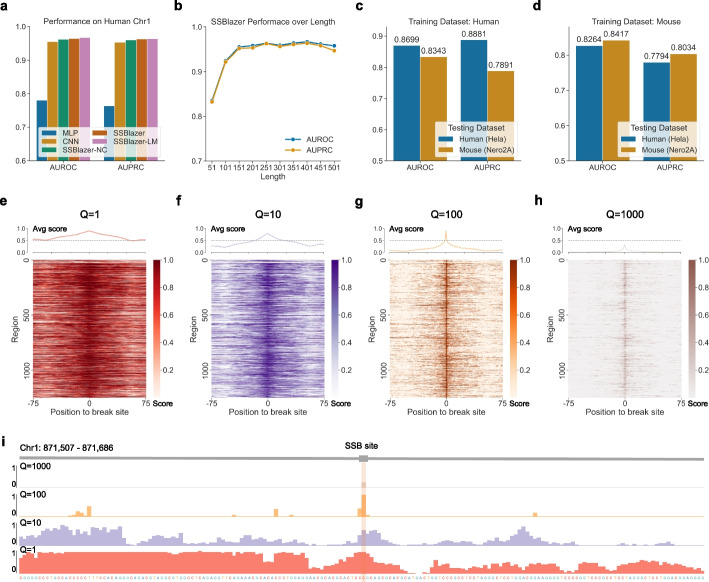
Model performance evaluation and the effect of different imbalance ratios in the dataset. **a** The model performance comparison of the proposed models (in AUROC and AUPRC). SSBlazer-NC refers to SSBlazer without the center feature processing module. SSBlazer-LM refers to a language model version of SSBlazer. **b** Assessment of AUROC and AUPRC values across varying input sequence lengths, ranging from 51 bp to 501 bp, to determine the optimal context length. **c**, **d** Cross-species evaluation reveals that SSBlazer exhibits desirable cross-species generalization ability. SSBlzer was first trained the model on dataset II-A (*Homo sapiens*) and evaluated the model performance on dataset II-B (*Mus musculus*) and then had the reverse experiment (II-B for training, II-A for evaluation). **e–h** Profile heatmaps on 1250 ground truth SSB sites illustrate the impact of introducing imbalanced datasets (*Q *= 1, *Q *= 10, *Q *= 100, and *Q *= 1000) on the 151 bp region around the SSB sites of the human genome (hg19) chromosome 1. These signal-to-noise landscapes reveal that the introduction of imbalances can sufficiently reduce false positives. **i** Prediction scores for a specific ground truth SSB site region (Human chr1: 871,507–871,686) of *Q *= 1 model (red), *Q *= 10 model (purple), *Q *= 100 model (orange), and *Q *= 1000 model (brown). The model trained on the balanced dataset shows a high false**-**positive rate in the flanking regions. The model trained with the imbalanced dataset (*Q *= 100) has a significantly narrow peak at the ground truth SSB site and a relatively low signal in the flanking regions

To assess the performance of SSBlazer, a leave-one-chromosome-out testing strategy was employed on the S1 END-seq dataset within the human neuronal cell line. Given the high sensitivity and robustness of the two sequencing methods [18, 19], negative samples were generated by random sampling, excluding the positive positions. The dinucleotide sampling [21] is also tested and included in the Supplementary (Additional file 1: Fig. S1). When evaluating the efficacy of dinucleotide-shuffled sequences in distinguishing SSB sites, we found that the model trained on such a dataset fails to capture the genomic occurrence pattern of SSBs. SSBlazer, being the first computational approach for SSB site prediction, was compared with two conventional baseline models (MLP and CNN), SSBlazer-LM (a language model version of SSBlazer), and SSBlazer-NC (SSBlazer without the center stream). These results suggest that SSBlazer can accurately predict SSB sites. The area under the receiver operating characteristic (AUROC) and the area under precision-recall curve (AUPRC) were employed to measure the prediction performance of the different models (refer to the “Methods” section for more details). Figure 2a shows that SSBlazer significantly outperforms the baseline machine learning models (CNN and MLP) in both AUROC (MLP = 0.7808, CNN = 0.9548, SSBlazer = 0.9626) and AUPRC (MLP = 0.7640, CNN = 0.9530, SSBlazer = 0.9621). This indicates that SSBlazer can efficiently capture the pattern of SSB sites and has a superior capacity to distinguish genuine SSB sites. Furthermore, SSBlazer achieved comparable performance to the BERT-based language model SSBlazer-LM with only 1.7% of the parameters (SSBlazer-LM: 110M, SSBlazer: 1.9M), for both AUROC (SSBlazer = 0.9626, SSBlazer-LM = 0.9661) and AUPRC (SSBlazer = 0.9621, SSBlazer-LM = 0.9634), thereby laying the foundation for large-scale downstream applications.

An ablation study was carried out to examine the contribution of the center feature. As seen in Fig. 2a, omitting the center feature led to a slight decrease in model performance (AUROC by 0.41% and AUPRC by 0.53%), emphasizing the importance of the center context. In addition, the impact of various lengths of SSB site context on the model was investigated. For this purpose, variants of the S1 END-seq dataset were created with different sequence lengths ranging from 51 bp to 501 bp to determine the optimal context length. Figure 2b illustrates that models with a context length over 251 bp achieved superior performance in both AUROC and AUPRC. This suggests that SSBlazer is capable of capturing long-range dependencies and utilizing dense context information to enhance model capacity. In this study, the model was constructed using a 251 bp sequence length. This length was chosen because the model with 251 bp achieves highly comparable performance and reduce model complexity compared to models with longer sequence lengths.

### SSBlazer exhibits robust cross-species generalization ability

The cross-species ability of bioinformatics tools is an indispensable component of modern biological research, offering a powerful means to study organisms that are inherently difficult to access or experimentally manipulate [23]. By leveraging existing genomic data, researchers can explore the genetic landscapes of inaccessible species and unravel the evolutionary forces shaping their genomes *in silico*. This ability holds tremendous promise to unlock new biological insights and inform biomedical research across a broad spectrum of species. To this end, we conducted a cross-species benchmarking to evaluate the extensive applications of SSBlazer across diverse species. Since the S1 END-seq dataset only covered *Homo sapiens*, we integrated two datasets for *Homo sapiens* and *Mus musculus*, which were generated by the SSiNGLe-ILM [19] approach, for cross-species evaluation. Initially, the model was trained on dataset II-A (*Homo sapien*s) and its performance was evaluated on dataset II-B (*Mus musculus*). We then conducted a reverse experiment, training on II-B and evaluating on II-A. Figure 2c, d show that the model trained on the human dataset also achieved comparable performance on the mouse genome (AUROC: 0.8699, 0.8343; AUPRC: 0.8881, 0.7891), and the reverse experiment yielded a consistent conclusion (AUROC: 0.8264, 0.8417; AUPRC: 0.7794, 0.8034). These observations suggest that SSBs in different species may share similar genomic patterns, and SSBlazer can capture such patterns. Thus, SSBlazer demonstrates a substantial cross-species generalization ability, making it feasible for further cross-species applications.

### Imbalanced dataset reduces false positive prediction

Several studies [18, 19] revealed that SSB sites are rarely distributed in the genome and follow a specific pattern. Therefore, constructing a dataset with an imbalanced positive-negative distribution more accurately simulates the real-world scenario, where negative samples outnumber positive ones (refer to the “Methods” section for more details). To investigate the impact of the imbalance ratio *Q*, we trained the model with different datasets (*Q *= 1, 10, 100, and 1000). We conducted a genome-wide analysis to discern the model’s signal-to-noise status across different imbalance ratios. The profile heatmaps of 1250 ground truth SSB sites (Fig. 2e–h) illustrate the signal-to-noise landscapes with different imbalanced ratios. These heatmaps reveal that most of the models (*Q *= 1, 10, and 100) can successfully identify the genuine SSB sites in the center region. The model training on the balanced and mildly imbalanced datasets (*Q *= 1, 10, Fig. 2e, f) have a relatively high signals on the flanking sequences. Notably, the model with *Q *= 100 (Fig. 2g) shows a clear signal at the center region, indicating that the introduction of an imbalanced dataset can sufficiently reduce false positive prediction on flanking sequences. However, the model training on the extremely imbalanced dataset (*Q *= 1000) is struggling to locate many of the true SSB sites. The model training on the imbalanced dataset (*Q *= 100) can precisely distinguish the genuine SSB sites and efficiently alleviate false positive predictions. Thus, we employed this dataset for further model construction. Specifically, the predicted scores on the genomic region with a ground truth SSB site (chr1: 871,507–871,686) were visualized by the Integrative Genomics Viewer (IGV, Fig. 2i). This visualization revealed that increasing the imbalanced ratio *Q* can effectively mitigate false positive predictions. Moreover, the model trained on the imbalanced dataset (*Q *= 100) presents a significantly narrow and pointed peak at the actual SSB site, leading to an accurate enrichment of the predicted SSB site into the genuine site. These results indicate that training SSBlazer on an imbalanced dataset can efficiently reduce false positives, and the predicted SSB frequency more accurately corresponds to the realistic genome-wide distribution. This provides a viable approach to portraying genome vulnerability.

### SSBlazer captures the pattern of CpG dinucleotides

**Fig. 3:**
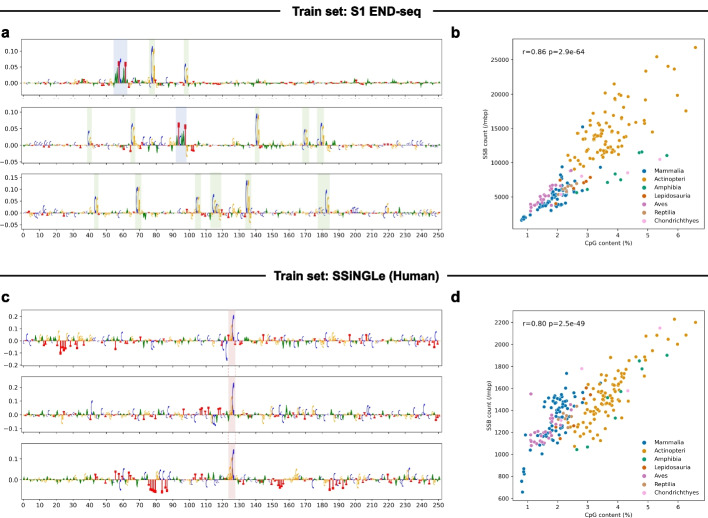
SSBlazer captures specific motif patterns of the SSB site context and determines the crucial role of CpG dinucleotide. **a** Integrated gradients analysis in S1 END-seq dataset reveals contribution scores of ground truth SSB site context. The individual CpG pattern is discontinuously distributed in the SSB site context, and the motifs of CCG and CGG are disfavored in SSB site context. The motif of ATCAAT also arises in the SSB site context in S1 END-seq dataset. **b**,** d** Correlation analysis demonstrated the association between CpG content and predicted SSB counts in 212 species using Pearson correlation test (S1 END-seq: *r* = 0.86, *p* = $2.9 \times e^{-64}$, SSiNGLe-ILM: *r* = 0.80, *p* = $2.5 \times e^{-49}$). **c** Contribution scores in the SSiNGLe-ILM dataset discover an explicit motif of GGC in the center region of the SSB site sequence context

To understand the SSB occurrence mechanism via the explainable model, we utilized the integrated gradients method by Captum [24] to determine which nucleotide decides the prediction outcome. The integrated gradients method quantifies each nucleotide’s impact on the model’s prediction by integrating its output gradient, revealing the influence of input variations on the prediction. We visualized the estimated contribution scores on the individual putative sequences [22] (Fig. 3a, c), and the direction of the nucleotide represents the contribution to the classification. If the nucleotide is above the *x*-axis, it positively contributes to the sequence being classified as a single-strand break site and vice versa. Among the sequences predicted to be SSB sites in S1 END-seq (Fig. 3a), CpG dinucleotides are observed to receive significant contribution scores. CpG dinucleotides are specific DNA sequences characterized by the presence of a cytosine (C) followed by a guanine (G) nucleotide, connected by a phosphate group [25]. These dinucleotides serve as pivotal elements in epigenetic regulation, exerting influence over the stability of the genome by modulating DNA repair processes [26]. We observed an enhanced frequency of CpG dinucleotides in an SSB site context, which is consistent with the genome-wide landscape that SSBs are more likely to be found at or near CpG dinucleotides [18]. Interestingly, the CpG pattern is discontinuously distributed, and the upstream cytosine and downstream guanine of CpG contribute negatively to putative positive SSB site prediction outcome, indicating that CCG and CGG trinucleotides are disfavored in the SSB site sequence context. In addition, the motif of ATCAAT also arises in the SSB site context. Furthermore, we also conducted the interpretation analysis of the SSiNGLe-ILM dataset (Fig. 3c). An explicit motif of GGC can be found in the center region of the SSB site context, indicating the distinct genomic pattern of that in S1 END-seq dataset, which can be explained by the bias from different SSB detection approaches and cellular states.

As mentioned above, CpG dinucleotides are an important pattern in SSB site recognition. In addition, SSBlazer exhibits robust cross-species generalization ability. Thus, we performed a correlation analysis to determine the association between CpG content and putative SSB numbers in different species. The average content of CpG dinucleotides was obtained from normalized sampling to remove the bias from the diverse genome sizes of different species. We randomly collected 1 million sequences of each species with a length of 251 bp from chromosome 1 and calculated the average CpG counts. It is worth noting that the correlation analysis in both S1 END-seq and SSiNGLe-ILM datasets shows a significant relationship between average CpG contents and SSB counts among various species. Figure 3b, d reveal that the number of SSB increases in the species with higher CpG content and has a strong positive correlation with the CpG content (Fig. 3b, d, Pearson correlation test, S1 END-seq: *r* = 0.86, *p* = $2.9 \times e^{-64}$, SSiNGLe-ILM: *r* = 0.80, *p* = $2.5 \times e^{-49}$), indicating that SSBlazer can sufficiently capture the pattern of CpG. The simultaneous analysis was conducted on chromosomes 2 and 3 and the whole genome (Additional file 1: Fig. S2), and the results revealed similar trends. Besides, several studies [27, 28] have found that a possible source of DNA lesion is cytosine methylation/demethylation in the CpG region, which is consistent with the interpretation analysis. Thus, the explainable model of SSBlazer may provide novel potential insights into the SSB occurrence mechanism, such as the recognition motif of ATCAAT and the break site motif of GGC.

### SSBlazer enables large-scale application with lightweight structure

The growing demand for computing resources in the field of deep learning often limits large-scale downstream applications. This study aims to depict the SSB genomic landscape of various species using a more efficient model. We demonstrate that our model, SSBlazer, achieves performance highly comparable to the BERT-based language model SSBlazer-LM but only requires 1.7% of the computing resources. Our experiment was conducted on 1 $\times$ NVIDIA A100 GPU with 80 GB memory using S1 END-seq dataset and the batch size as 2048. To qualify the occupied computing resources, MACs (multiply-accumulate operations) was utilized and obtained by thop v.0.0.31 (https://github.com/Lyken17/pytorch-OpCounter). The results indicate that SSBlazer saves 99% of the MACs and 98% of the parameters compared to SSBlazer-LM and maintains a comparable performance (AUROC reduced by 0.0035, AUPRC reduced by 0.0013). We further applied SSBlazer to the S1 END-seq dataset to ascertain the training and inference time. For SSBlazer, the training time for a single epoch is 30 s, and the inference time is 8 s. This is significantly less than that of SSBlazer-LM, which takes 422 s for training and 29 s for testing. Moreover, the usage of GPU RAM for SSBlazer is only 6,971 MB with the current batch size, significantly lower than that of SSBlazer-LM (46,287 MB). This suggests that SSBlazer could further increase the batch size to fully utilize computational resources and enhance the training and inference process. In conclusion, these results demonstrate that SSBlazer is a lightweight model that enables large-scale parallel inference to depict SSB landscapes on diverse genomes (Table 1).

**Table 1:** Performance and computational complexity of SSBlazer and SSBlazer-LM. Each model was trained on 1 $\times$ NVIDIA A100 GPU with 80 GB memory, and the batch size was 2048. Training time refers to the backpropagation time of the training set of S1 END-seq dataset (imbalance ratio = 1, number of samples = 231,500), and inference time is the forward propagation time during the testing period (number of samples = 22,044)

Model	AUROC	AUPRC	MACs	Params	Training (s)	Inference (s)
SSBlazer-LM	0.9661	0.9634	43661.70G	110M	422	29
SSBlazer	0.9626	0.9621	332.77G	1.9M	30	8

### SSBlazer web server

**Fig. 4:**
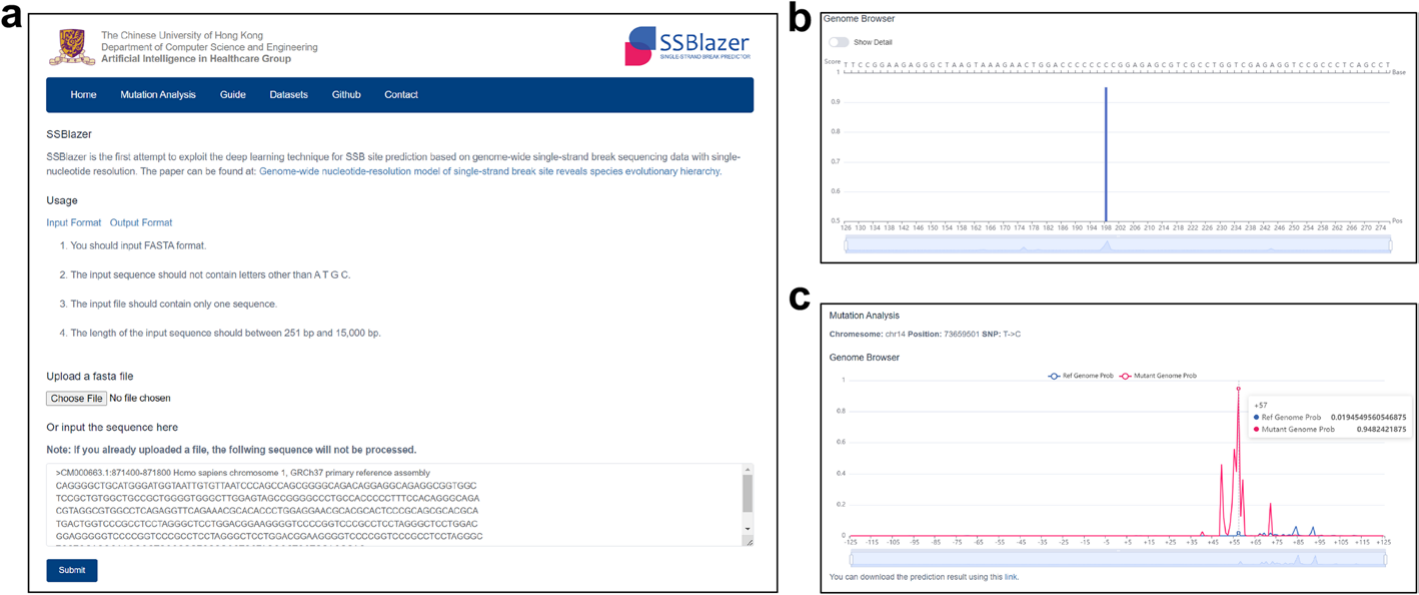
SSBlazer web server. **a** Screenshot of SSBlazer web server interface. The users can submit a single sequence up to 15,000 bp in fasta file or input the DNA sequence directly into the text box. **b** The visualization of input sequence SSB score for each putative SSB site position. We also provide a toggle for SSB scores of the whole query sequence instead of the putative SSB sites. **c** The mutation analysis profiled alterations in the SSB landscape, identifying SSB-related SNPs

The accumulation of SSBs has been associated with multiple diseases. However, there has been a lack of in silico tools for SSB site prediction. In this study, we have depicted vast landscapes of SSB sites for diverse species via SSB frequency using SSBlazer, but there are still many unexplored SSB-related applications for this tool. One intriguing possibility is investigating how changes in the SSB landscape within a genomic region can provide valuable insights into the mechanisms of how the host gene or nearby SNP contributes to disease progression. To illustrate, we conducted a SNP comparison analysis on Alzheimer’s disease (AD) patients. Emerging evidence suggests that genetic factors, including SNPs, play a critical role in AD susceptibility [29, 30]. However, specific mechanisms of SNPs in AD development are still being understudied. In this study, we collected pathogenic SNP data from ClinVar [31] and applied SSBlazer to assess how such genomic substitutions might influence the local SSB landscape. Our findings (Fig. 4c) indicate that a particular SNP (rs123456) in ClinVar alters the SSB landscape against the reference genome, suggesting that pathogenic SNPs may promote the occurrence of AD via SSB-related pathways. To facilitate usage, we have established the web server (Fig. 4a) for intensive downstream applications. Users only need to provide a query sequence, and SSBlazer will process it automatically, outputting a prediction score for each position in the sequence (Fig. 4b). The web server also provides the option to release scores for the entire sequence rather than just the putative SSB sites. Additionally, the processed dataset used in SSBlazer is available for the enhancement of model construction. The new SSB dataset can be easily incorporated into SSBlazer to alleviate detection approach bias. The web server of SSBlazer is now available at https://proj.cse.cuhk.edu.hk/aihlab/ssblazer/. The future version will expand the species and genomic annotation features, providing an even more comprehensive tool for SSB analysis.

### SSBlazer explores SSB-related applications: case study

**Fig. 5:**
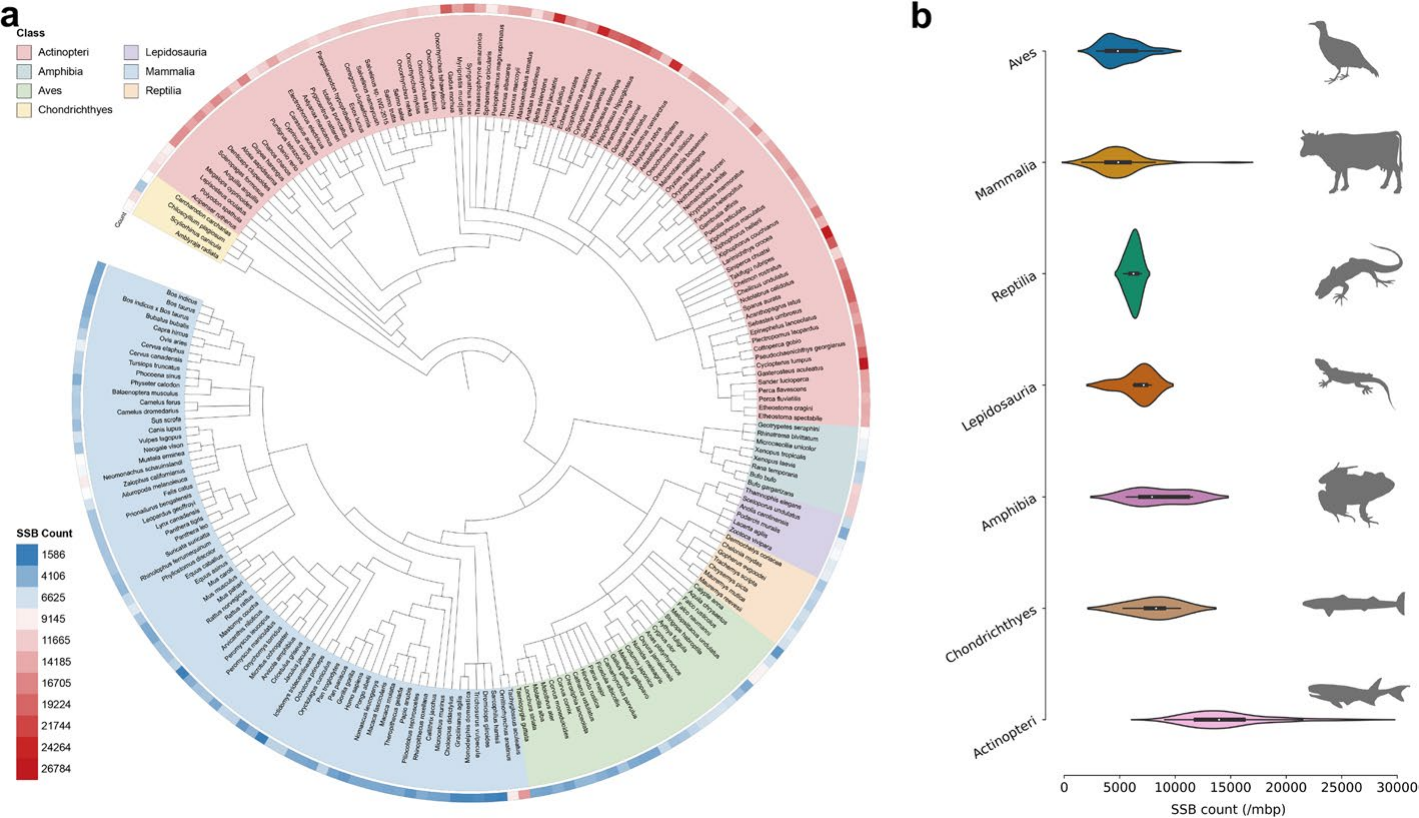
SSB frequency corresponds to species evolutionary hierarchy. **a** The phylogenetic diagram on 212 vertebrates indicates their evolutionary hierarchy, and the peripheral heatmap represents the number of SSBs. **b** Violin plots suggest order differences in SSB counts ranked by mean counts. Silhouettes of animals are downloaded from PhyloPic (http://www.phylopic.org)

The successful construction of SSBlazer has enabled a plethora of applications related to SSBs. SSB frequency is considered an implicit feature of genomic integrity [32], which could be linked to differences in the evolutionary process across diverse species. Cross-species evaluation in human and mouse datasets indicated that SSBs across diverse species might share similar genomic patterns. SSBlazer accurately captures these patterns, demonstrating robust cross-species generalization ability. Thus, we first collected various vertebrate reference genomes (212 non-redundant species including* Actinopteri, Mammalia, Reptilia, Lepidosauria, Chondrichthyes, Amphibia, Aves.*) from the Genbank [33] and RefSeq [34]. Given the biases that might arise due to varying genome sizes, we employed normalized sampling. Specifically, we generated 1 million sample sequences, each with 251 bp, from chromosome 1 of each reference genome [35]. Finally, we applied SSBlazer to this large collection of sequences to identify putative SSB sites. This allowed us to estimate the SSB frequency and, consequently, the genome integrity of each species. Remarkably, the predicted frequency of SSB is highly variable among different species, ranging from 1586 in thirteen-lined ground squirrel (*Ictidomys tridecemlineatus*) to 26,784 in Alaskan stickleback (*Gasterosteus aculeatus*), and a clear trend along the evolutionary process is observed. The phylogenetic diagram (Fig. 5a) and the violin plot (Fig. 5b) indicate the SSB count distribution at the species and class level. These results reveal that *Actinopteri* and *Chondrichthyes* may have relatively high levels of SSB frequency, while *Reptilia*, *Mammalia*, and *Aves* share fewer SSB frequencies. This broad analysis provides a comprehensive view of predicted SSB frequency across a diverse range of vertebrates, shedding light on its role in genomic integrity and evolutionary processes.

## Discussion

High-throughput SSB detection assays have emerged recently and provided approaches for describing the SSBreakome generated by the direct action of several genotoxins or common intermediate products of DNA transactions [4]. The landscapes of genome vulnerability derived from various cell lines can characterize the genome in various aspects such as evolutionary hierarchy, animal phenotype, and cancer development. However, while the NGS-based methods led to the discovery of SSBreakome, these approaches are costly and highly reliant on advanced sequencing equipment. This makes it difficult to perform large-scale applications on diverse species. Furthermore, despite sharing a similar SSB detection strategy, method bias exists in constructing the SSBreakome landscape. To alleviate this bias, a unified and comprehensive SSB detection schema should be proposed in the future. The current version of SSBlazer was trained on the S1 END-seq dataset and the SSiNGLe-ILM dataset. While SSBlazer is a portable and well-organized model, which can smoothly transition to the unified dataset and other available SSB detection datasets (e.g., SSB-seq [36], GLOE-seq [20]). We provided a retraining tutorial in https://sxu99.gitbook.io/ssblazer/use-cases/train-a-new-model. This adaptability can substantially improve SSBlazer’s generalization ability, making it a valuable tool in the continued exploration and understanding of the SSBreakome.

In this study, we proposed a deep learning-based framework to predict SSB sites by integrating the underlying genomic context. To our knowledge, SSBlazer is the first attempt to exploit a computational model for SSB site prediction based on genome-wide single-strand break sequencing data and furthermore at a single-nucleotide resolution. We demonstrated that SSBlazer could accurately identify SSB sites and exhibit robust cross-species generalization ability. The introduction of the imbalanced dataset simulated the realistic SSB distribution in the genome and sufficiently reduced false positives. Notably, the interpretation analysis revealed that SSBlazer captures the pattern of individual CpG in the genomic context and the motif of GGC in the center region as critical features and also provides the hypothesis for SSB occurrence mechanism exploration such as the recognition motif of ATCAAT in the SSB site context. Since SSBlazer is a lightweight model with robust cross-species generalization ability in cross-species evaluation, it has been successful in various applications related to SSBs. In a specific case study, SSBlazer was employed on a sample of 212 vertebrate genomes, yielding intriguing putative SSB genomic landscapes. These landscapes offered a captivating proposition regarding the potential correlation between the frequency of SSBs and the hierarchy of evolution. Moreover, SSBlazer represents a valuable tool for delineating disease conditions. It can illustrate the alterations in the SSB landscape attributed to mutational events as well as characterize the properties of pathogenic SNPs that induce SSB. A recent study [37] conducted genome-wide characterization of DNA microsatellite repeats in fish and found that the frequency of DNA microsatellite repeats plays a vital role in chromatin organization, recombination, and DNA replication. This suggests a correlation between the abundance of DNA microsatellite repeats and the high level of SSBs in *Actinopteri* and *Chondrichthyes*. Additionally, a DSB detection approach called Breaks Labelling, Enrichment on Streptavidin, and next-generation Sequencing (BLESS) also found the enrichment of DSBs located in the microsatellite repeats region [14]. These results demonstrated that such DNA lesions are non-randomly located in the genome and have specific distribution patterns. SSBlazer can distinguish the genomic pattern of SSB and may bring novel molecular insights into the physiological and pathological progress.

Despite the desirable performance of SSBlazer, further work is required to enhance its capabilities. Currently, SSBlazer employs only the DNA context as input information. This means that the model cannot precisely characterize the species’ genome and differentiated cellular states due to the loss of genomic annotation, such as genomic region, structure, and epigenetic information. For example, the study [19] has revealed the enrichment of SSBs at the region of exons and other transcriptional regulatory elements such as CTCF binding sites. In addition, a DSB prediction model [38] demonstrated the use of the DNA structural information such as minor groove width (MGW), propeller twist (ProT) at base-pair resolution, roll (Roll), and helix twist (HelT). This information can efficiently improve the model’s performance and reveal the crucial role of DNA structural information in lesion recognition. In CRISPR-induced DSB prediction [39, 40], the epigenetic landscapes (e.g., CTCF, Dnase, H3K4me3, and RRBS) are regarded as additional features of the model. The ablation experiment also reveals the importance of such chromatin status markers in the CRISPR-induced cleavage process. Therefore, future versions of SSBlazer should consider incorporating additional genomic information, such as genomic region, structure, and epigenetic information. This will allow the model to more accurately characterize the vast and unique SSB genomic landscape in diverse species’ genomes, differentiated cellular states, and distinct development stages. Our study has several limitations. A primary limitation is the current reliance on human data for validation, utilizing available ground truth datasets. While the complexity of the human genome provides a strong foundation for evaluating prediction performance, we recognize the need for broader validation across different species. Additionally, the absence of an outgroup control species is an important consideration. Despite these limitations, we believe our current findings still contribute to the understanding of SSB patterns and the potential applications of our tool.

The genome landscape of the SSB sites reveals the association between DNA lesions and various cellular conditions. Therefore, landscapes of genome vulnerability derived from the various cell lines and phenotypes may contribute to previously unutilized insight for developing molecular biomarkers of disease diagnosis, aging identification, and gene therapy. Specifically, SSBs could lead to the accumulation of somatic mutations and transcription stalling in functional neuronal genes, contributing to neurological diseases. These diseases have been reported by association with defective SSB repair systems [41]. In addition, DNA lesions have been widely implicated in human aging, and a recent study [19] emphasized the association between SSBreakome patterns and chronological age in humans. Understanding the landscape of genome vulnerability can also aid in the design of gene therapy strategies. For instance, knowing the target site’s vulnerability landscape can guide the design of an optimal gene editing approach (e.g., high-efficiency and high-fidelity sgRNA), potentially improving gene therapy outcomes. This SSB landscape may also offer insights into the recognition and cleavage mechanism in the CRISPR/Cas9 system. SSBlazer has demonstrated satisfying performance and strong cross-species generalization ability. The putative genomic landscape of the SSB site characterized by SSBlazer on diverse species may shed light on the mechanisms of aging and complex diseases in various animal models.

## Conclusions

 

- SSBlazer is a significant advancement in the field of computational genomics. It represents the first computational approach capable of genome-wide nucleotide-resolution SSB site prediction. SSBlazer can accurately identify SSB sites with only the context sequence as input.
- SSBlazer is an interpretable model. It reveals individual CpG dinucleotides as the crucial feature, consistent with a previous study. Besides, SSBlazer also captures previously undiscovered recognition motifs and break site motifs.
- SSBlazer provides an intuitive web server, poised to address a multitude of unexplored challenges related to single-strand breaks, such as the analysis of pathogenic SNP mutations.
- SSBlazer elucidates the conjectural genomic landscapes of single-strand breaks across all accessible vertebrate genomes in the cross-species analysis. Remarkably, the prospective SSB genomic landscapes derived from a study of 212 vertebrates demonstrate a potential hypothesis about the correlation between SSB frequency and the respective evolutionary hierarchy, which needs to be further experimentally validated.

## Methods

### Dataset construction

#### Data source

##### Dataset I

The S1 END-seq data of human i^3^Neurons cell line was collected from the study of Wu et al. [18]. This data was processed using the standard bioinformatics pipeline for END-seq [42]. The initial step involved downloading the original sequencing data from the Gene Expression Omnibus (GEO) database (GSE167259) and collecting the raw reads of the ddN S1 END-seq sample via the SRA Run Selector. Quality control was subsequently conducted using FastQC (v.0.11.9), and Trim Galore (v.0.6.4) was employed to remove adapters and low-quality reads. The clean reads of the END-seq were then aligned to the human genome (hg19) using bowtie (v.1.1.2), and a sorted bam file was created with the aid of samtools (v.1.14). After genome alignment, peak calling was performed with MACS2 (v.2.2.7.1), and the peak summits were gathered to generate SSB sites (positive sample). Given the summit coordinate, we extracted the 125 bp sequence context upstream and downstream from the human genome (hg19) to enhance performance, resulting in a final input sequence length of 251 bp. Furthermore, to augment the data, we introduced a reverse complementary sequence for each positive sample, as S1 END-seq is a non-strand-specific SSB detection approach. The final positive dataset comprised 126,772 sequences (Table 2).

**Table 2:** Datasets of single-strand break sites. Dataset I is reconstructed by the standard bioinformatics pipeline of END-seq analysis. Dataset II is provided in the original paper of SSiNGLe-ILM [19] and is established to evaluate generalization ability and cross-species performance

Index	Cell lines	Methods	Validated SSB sites	Strand-specific	Assembly	Reference
I	i^3^Neurons	S1 END-seq	63,386	No	hg19	[18]
II-A	HeLa	SSiNGLe-ILM	2,331,388	Yes	hg19	[19]
II-B	Neuro2A	SSiNGLe-ILM	275,432	Yes	mm10	[19]

##### Dataset II

To assess the generalization ability and cross-species performance of the model, we established the SSiNGLe-ILM dataset. This dataset includes data from the Hela cell line (*Homo sapiens*, II-A) and the Nero2A cell line (*Mus musculus*, II-B), as provided by Cao et al. The integral SSB sites for Hela (GSM4126203) and Nero2A (GSM4126206) were extracted from the GEO database. Similar to dataset I, the 125 bp sequence context upstream and downstream from the SSB site coordinate was extracted from the respective genomes (Human genome hg19, mouse genome mm10), and the final length of each sequence is 251 bp. However, since SSiNGLe-ILM provides a strand-specific insight, the sequence context of SSB sites was extracted based on the specific strand, without the addition of reverse complementary sequences (Table 2).

#### Imbalanced dataset

The construction of a negative set often determines the practicality of the model in scientific applications. In an attempt to realistically simulate scenarios where SSB sites are sparsely distributed within the genome, we opted for an imbalanced dataset (where negative samples outnumber positive ones) instead of the traditional balanced dataset. This strategy is designed to compel the model to more precisely distinguish genuine SSB sites. In this context, we introduce the imbalance ratio, denoted by *Q*. For a given *N* positive sample, the number of negative sequences is $N \times Q$. To explore the impact of dataset imbalance on model performance, we constructed datasets with varying degrees of imbalance, using $Q=1,10,100$ and 1000. Negative sequences were randomly extracted from the reference genomes (hg19 and mm10), excluding the positive SSB sites, and each sequence was maintained at a length of 251 bp. For the construction of the final dataset, we implemented a leave-one-chromosome-out testing strategy [21]. In this arrangement, chromosome 1 was designated for testing, while the remaining chromosomes were used for training.

### Model framework

#### Data encoding

In SSBlazer, the sequence context is encoded as a 4 $\times$ *L* matrix using one-hot encoding, where each nucleotide in the sequence is converted into a binary vector, with each dimension corresponding to a nucleotide channel *A, C, G* and *T* for the following convolution operation. Formally, given a DNA sequence $s=(s_{1},s_{2},s_{3}, ...,s_{n})$ with *L* nucleotides, the one-hot encoding matrix *M* is:

$$\begin{aligned} M_{i,j}= \left\{ \begin{array}{ll} 1&{} \text {if}\ s_i=D_j\\ 0&{} \text {otherwise} \end{array}\right. \end{aligned}$$

where $s_i$ is the $i^{th}$ nucleic acid of the sequence and $D =[A, C, G, T]$.

Since SSBlazer-LM is a DNABERT-based language model pre-training on the human genome (GRCh38.p13), SSBlazer-LM takes *k*-mer tokens as the inputs, where *k*-mers are substrings with defined length *k* of a sequence. Given a sequence of length *L*, the set of *k*-mer tokens is constructed by dividing the sequencing with the stride of 1, and the number of *k*-mers is $L-k+1$. In this study, we used 6-mer representations for enlarging sequence context receptive recognition to improve model classification capability.

#### Model construction

##### SSBlazer

The model takes one-hot representations of the 251 bp sequence as the input and processes the whole sequence and the center 9 bp separately. We use a residual-based neural network to obtain the vector representation of the input sequence for the feature extraction. Inspired by ResNet [43], SSBlazer (Fig. 1b) starts with a 1 $\times$ 3 convolutional layer and follows by two sets of residual blocks with different convolution kernels. Each residual unit in the residual block consists of a 1 $\times$ 5 convolutional layer and a 1 $\times$ 3 convolutional layer. For the first 15 blocks, the number of filters $N_{f1}$ is 16, and $N_{f2}$ is 32 for the last 15 blocks. To alleviate the gradient vanishing problem, we apply the exponential linear unit (ELU) [44] as the activation function instead of the rectified linear unit (ReLU) [45]. After a 1 $\times$ 5 average pooling, the sequence feature is then passed to a multi-head self-attention layer to capture long-range dependencies:

$$\begin{aligned} MultiHead(M)= & {} Concat(head_1,...,head_h)W^O \\ head_i= & {} Softmax\left( \frac{MW_i^Q {MW_i^K}^T}{\sqrt{d_k}}\right) MW_i^V \end{aligned}$$

where $W^O \in \mathbb {R}^{hd_v \times d_{model}}$, $W_i^Q \in \mathbb {R}^{d_{model} \times d_k}$, $W_i^K \in \mathbb {R}^{d_{model} \times d_k}$ and $W_i^V \in \mathbb {R}^{d_{model} \times d_v}$ are learned parameter matrices for projection.

In this model, we employ 8 heads ($h=8$) and set $d_k=d_v=512$. $d_{model}$ depended on the input matrix ($d_{model}$ = 64 in our cases). Nucleotides around the SSB site are considered an important pattern in several studies [18, 27, 28]. Thus, we collect the center sequence as the extra input to capture the specific pattern of the center region. Due to the concise DNA sequence of the center region, models with high complexity (e.g., ResNet) tend to be overfitting. Therefore, we introduce a single-layer inception module [46] to enrich the center information. The inception module contains three parallel convolutional layers, and the kernel dimensions are 1 $\times$ 1, 1 $\times$ 3, and 1 $\times$ 5 separately. The outputs of these layers are concatenated and combined with the original one-hot matrix of center sequence. Finally, the sequence context and the center feature are concatenated and fed into the multilayer perception with two hidden layers for classification. Each fully connected layer is followed by a dropout with the ratio of 0.5 and 0.3 separately to improve the generalization ability.

#### Model interpretation

To interpret the proposed model intuitively, we performed the integrated gradient analysis [24] to illustrate how the individual nucleic acid influences the prediction outcome and determine potential motifs of predicted SSB sites. In contrast to extracting motifs from the putative SSB site context sequences, the integrated gradient analysis is a comprehensive gradient-based axiomatic attribution method that computes a contribution score for each input feature compared to a baseline. It can efficiently visualize the genomic pattern that the model captures. In this study, integrated gradient analysis emphasizes the contribution score of each nucleotide in the sequence containing an SSB site. Formally, $x \in \mathbb {R}^n$ is the input sequence, $x' \in \mathbb {R}^n$ is the baseline sequence, and $F: x \in \mathbb {R}^n \rightarrow <span class='reftype'>[0, 1]</span>$ is the deep learning model; integrated gradients are calculated by accumulating the gradients $\frac{\partial F(x)}{\partial x_i}$ along the path from the baseline input $x'$ and the positive sample input *x*, where $x_i$ refers to the $i^{th}$ feature:

$$\begin{aligned} IntegratedGrad\quad s_i(x) :{:=}(x_i-x'_i) \times \int _{\alpha =0}^{1} \frac{\partial F(x'+\alpha \times (x-x'))}{\partial x_i} d\alpha \end{aligned}$$

The baseline input $x'$ should be a neutral input for the model, i.e., the prediction of it should be close to zero ($F(x)\approx 0$). In this research, we obtained the baseline sequence based on human GC content. Therefore, in the one-hot encoding matrix for the baseline sequence, each column is [0.3,0.2,0.2,0.3], representing the appearance probability of *A*, *C*, *G*, and *T*.

### Evaluation metrics

In this study, we have chosen AUROC and AUPRC as our evaluation metrics due to their effectiveness in measuring the performance of classification models:

$$\begin{aligned} \text {AUROC}= & {} \int _{0}^{1} \text {TPR}(t)\,d\text {FPR}(t) \\ \text {AUPRC}= & {} \int _{0}^{1} \text {Precision}(r)\,d\text {Recall}(r) \end{aligned}$$

Both of these metrics provide an aggregate measure of performance across all possible classification thresholds. The AUROC is particularly useful as it is invariant to the class distribution or the threshold setting. The AUROC ranges from 0 to 1, with a value of 0.5 indicating a random model and a value of 1 indicating a perfect model. The higher the AUROC, the better the model’s performance at discriminating between the positive and negative classes. AUPRC is based on precision and recall. Precision measures the proportion of true positive predictions among all positive predictions, while recall measures the proportion of true positive predictions among all true positive instances. AUPRC is especially useful when the cost of false positives and false negatives is different, as it allows for the identification of the optimal classification threshold that minimizes the cost.

### Construction of SSB site landscape in a species-wide manner

After the construction of SSBlazer, we depicted vast landscapes of SSB sites species-wide. We first collected all the available genomes of vertebrates (212 reference genomes) from the Genbank and RefSeq database using python scripts (https://github.com/kblin/ncbi-genome-download). The scripts originally obtained 220 reference genomes. After the removal of the duplicates and classes that only contain one or two species, there are 212 species in total, including 89 species in *Actinopteri*, 72 species in *Mammalia*, 26 species in *Aves*, 8 species in *Amphibia*, 7 species in *Reptilia*, 6 species in *Lepidosauria*, and 4 species in *Chondrichthyes*. To remove the bias of various genome sizes, we employed normalized sampling, where we randomly generated one million sequences with 251 bp from chromosome 1 of each reference genome. We applied SSBlazer on these candidates for identifying SSB sites and introduced SSB frequency to estimate the genome integrity. To visualize the evolutionary relationship among the 212 species, we employed iTOL (https://itol.embl.de/) to illustrate the evolutionary phylogenetic tree of these vertebrates based on species taxonomy ID. We integrated the SSB frequency heatmap into the evolutionary phylogenetic tree to discover the association between SSB frequency and evolutionary hierarchy.

### Supplementary Information

**Additional file 1:** Including Supplementary Texts, Supplementary Tables S1-S2 and Supplementary Figs. S1-S2. **Table S1.** Ablation for baseline MLP models. **Table S2.** Ablation for baseline CNN models. **Figure S1.** Performance Comparison Between Dinucleotide-Shuffle and Random Sampling Methods for SSB Site Discrimination. **Figure S2.** Correlation Between CpG Content and Predicted SSB Counts Across Different Chromosomal Regions.

**Additional file 2.** Peer review history.

## Data Availability

SSBlazer web sever is now available at https://proj.cse.cuhk.edu.hk/aihlab/ssblazer/ [47] for simplified usage. The source code of SSBlazer is available at https://github.com/sxu99/ssblazer; it is also hosted at here [48] for an archived version. The tutorial is available at https://sxu99.gitbook.io/ssblazer/. The dataset used in this study can be assessed at https://proj.cse.cuhk.edu.hk/aihlab/ssblazer/#/datasets/ [47] and also available here [47] The bioinformatics pipeline for the dataset construction of S1 END-seq can be found at https://www.ncbi.nlm.nih.gov/geo/query/acc.cgi?acc=GSM5100382 [48], and the processing scripts can be found at https://github.com/sxu99/ssblazer. All repositories are released under an MIT license.
