# Supplementary material for: SSBlazer: a genome-wide nucleotide-resolution model for predicting single-strand break sites
Source: Genome Biol. 2024 Feb 12;25:46. doi: 10.1186/s13059-024-03179-w (PMC10863285; doi:10.1186/s13059-024-03179-w)
Supplement: Supplementary file 1 — Additional file 1: Including Supplementary Texts, Supplementary Tables S1-S2 and Supplementary Figs. S1-S2. Table S1. Ablation for baseline MLP models. Table S2. Ablation for baseline CNN models. Figure S1. Performance Comparison Between Dinucleotide-Shuffle and Random Sampling Methods for SSB Site Discrimination. Figure S2. Correlation Between CpG Content and Predicted SSB Counts Across Different Chromosomal Regions. [file 13059_2024_3179_MOESM1_ESM.docx]

SSBlazer: A genome-wide nucleotide-resolution model for predicting single-strand break sites

***Supplementary Material***

**Baseline Models**

**SSBlazer-LM.** This model is a BERT-based language model, and all the parameters are initialized with those of DNABERT[1], which is a pre-trained language model on the human reference genome (GRCh38.p13). SSBlazer-LM uses k-mer representations of the DNA sequence as input tokens, where tokens are first generated from the input sequence and then converted into numeral IDs by a predetermined mapping. In this stage, token IDs are used to obtain token embeddings. Segment embeddings and position embeddings are determined by the sequence and the position of each nucleotide. All these embeddings are vectors with a length of 768, and these vectors are then fed into an element-wise addition module to generate the final embeddings of the input sequence. Then, these embedding vectors are processed by 12 transformer blocks to obtain the final context feature, and each transformer block consists of 12 attention heads and 768 hidden units. The final embedding output (E[CLS]) of the last hidden layer is regarded as the sequence feature and will be fed into the classification layer for prediction.

**MLP.** The MLP model uses a flattened vector of the one-hot encoded sequence as its input. From the ablation study, we have selected the MLP model with one hidden layer and a hidden dimension of 1024 as our baseline model, as it achieved the highest AUROC and AUPRC scores of 0.7808 and 0.7640 respectively (Table S1).

**CNN.** The input for the CNN model is identical to the SSBlazer, which is a 251×4 matrix. It is important to note that SSBlazer is also a CNN-based model. Therefore, we evaluated several fundamental CNN models as baselines for comparison. From our evaluations, the best-performing CNN model consisted of four convolutional layers, with the numbers of channels being [64, 64, 128, 128] and the kernel sizes being [3, 3, 3, 3]. This model yielded the highest AUROC and AUPRC scores of 0.9548 and 0.9530 respectively, and it has been chosen as our baseline CNN model (Table S2).

**Evaluating the Efficacy of Dinucleotide-Shuffled Sequences in Distinguishing SSB Sites**

We conducted an evaluation of an alternative negative sampling technique employed in DeepBind[2], where dinucleotide-shuffled sequences were used to generate negative samples. This technique ensures the preservation of all 16 dinucleotide counts (AA, AC, ..., GT, TT) from the original sequence, resulting in a higher sequence similarity. However, our findings revealed that the dinucleotide-shuffle method did not improve performance in differentiating SSB sites from the genomic background, compared to a dataset assembled with randomly sampled negative samples. On the contrary, the Area Under the Receiver Operating Characteristic (AUROC) exhibited a decline from 0.9626 to 0.8170, indicating a decrease in the predictive power of the model (Figure S1).

**Analyzing Chromosomal Conditions and Genome-Wide Correlations**

We pursued further research on the conditions of different chromosomes and their relationship with the whole genome. We conducted simultaneous analysis on chromosomes 1, 2, and 3 (Figure 3b, S2) and the results revealed similar trends (Chr1: r=0.86, p=2.9e^-64^; Chr2: r=0.87, p=1.6e^-62^; Chr3: r=0.84, p=7.9e^-38^). The correlation pattern obtained from whole-genome sampling was slightly different (Whole Genome: r=0.52, p=3.1e^-16^). We speculate that during the assembly process, the genomes of these insufficiently studied species contained some low- quality or unrecognizable chromosome data, which prevented the model from accurately identifying the SSB breakage frequency. Concurrently, the evaluation strategy of leave-chromosome1-out has been widely implemented.

**Table S1. Ablation for baseline MLP models.**

| Hidden Layers | Hidden Dimension | AUROC | AUPRC |
| --- | --- | --- | --- |
| **1** | **1024** | **0.7808** | **0.764** |
| 2 | 1024 | 0.7711 | 0.7599 |
| 3 | 1024 | 0.7723 | 0.7541 |
| 4 | 1024 | 0.7657 | 0.7453 |
| 5 | 1024 | 0.7655 | 0.7434 |

**Table S2. Ablation for baseline CNN models.**

| Conv Layers | Channels | Kernel Size | AUROC | AUPRC |
| --- | --- | --- | --- | --- |
| 2 | [32, 32] | [3, 3] | 0.9348 | 0.9300 |
| 3 | [32, 32, 64] | [3, 3, 3] | 0.9459 | 0.9425 |
| 4 | [32, 32, 64,64] | [3, 3, 3, 3] | 0.9532 | 0.9508 |
| 2 | [64,64] | [3, 3] | 0.9367 | 0.9319 |
| 3 | [64,64,128] | [3, 3, 3] | 0.9503 | 0.9471 |
| **4** | **[64,64,128,128]** | **[3, 3, 3, 3]** | **0.9548** | **0.9530** |


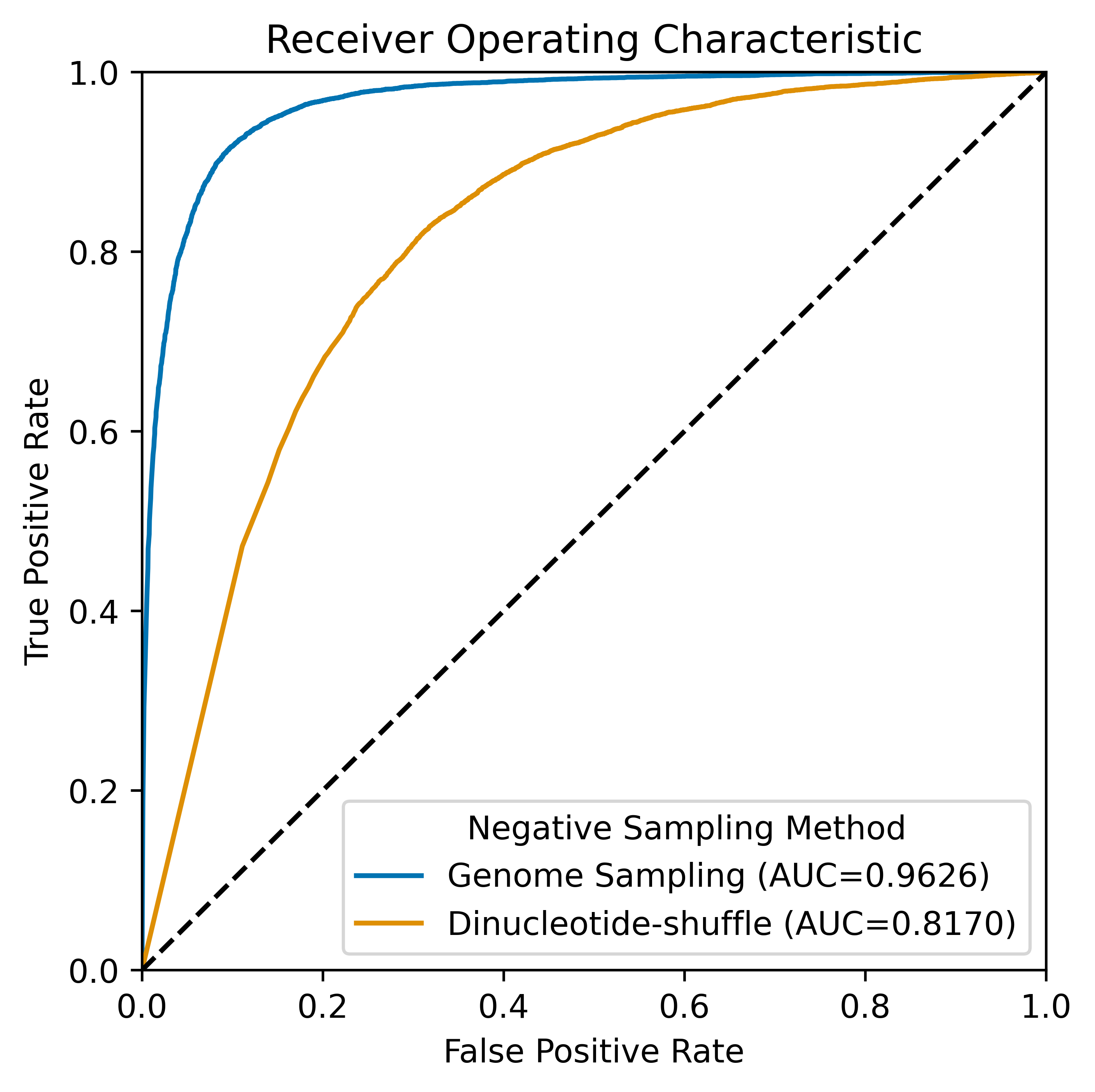


**Figure S1.** **Performance Comparison Between Dinucleotide-Shuffle and Random Sampling Methods for SSB Site Discrimination.** This figure illustrates the comparative performance of dinucleotide-shuffle generated negative samples versus randomly sampled negatives in differentiating single-strand break (SSB) sites from non-specific genomic background. It was found that the dinucleotide-shuffle method led to a decrease in the area under the receiver operating characteristic curve (AUROC), from 0.9626 to 0.8170. This indicates that the dinucleotide-shuffle technique is less effective for SSB site discrimination compared to the use of a randomly constructed negative sample dataset.


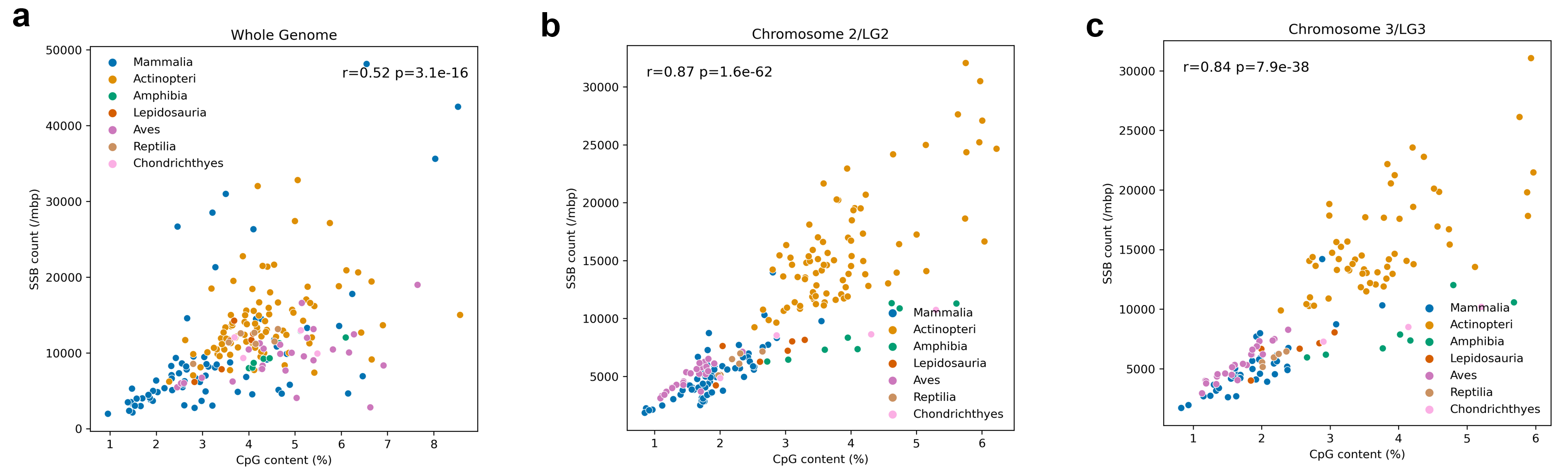


**Figure S2.** **Correlation Between CpG Content and Predicted SSB Counts Across Different Chromosomal Regions.** A correlation analysis was performed to explore the relationship between CpG content and predicted single-strand break (SSB) counts, focusing on chromosomes 2 and 3, as well as across the entire genome. The analysis indicated a strong positive correlation on chromosomes 2 and 3 (Chr2: r = 0.87, p = 1.6e^−62^; Chr3: r = 0.84, p = 7.9e^−38^), consistent with the trend observed on chromosome 1 (Figure 3 b, r = 0.86, p = 2.9e^−64^). In contrast, the correlation observed in the whole-genome analysis was moderate (Whole genome: r = 0.52, p = 3.1e^−16^), suggesting a divergence in the correlation pattern when assessed on a genome-wide scale.

**Reference**

1. Ji Y, Zhou Z, Liu H, et al. DNABERT: pre-trained Bidirectional Encoder Representations from Transformers model for DNA-language in genome[J]. Bioinformatics, 2021, 37(15): 2112-2120.
2. Alipanahi B, Delong A, Weirauch M T, et al. Predicting the sequence specificities of DNA-and RNA-binding proteins by deep learning[J]. Nature biotechnology, 2015, 33(8): 831-838.
