# Supplementary material for: SSBlazer: a genome-wide nucleotide-resolution model for predicting single-strand break sites
Source: Genome Biol. 2024 Feb 12;25:46. doi: 10.1186/s13059-024-03179-w (PMC10863285; doi:10.1186/s13059-024-03179-w)
Supplement: Supplementary file 2 — Additional file 2. Peer review history. [file 13059_2024_3179_MOESM2_ESM.docx]

**Review history**

**First round of review**

**Reviewer 1**

The manuscript by Xu and colleagues describes a new method to map genomic sites prone to single strand breaks using a flexible machine learning approach. This method is applied to the genomes of 216 vertebrates that have not had SSB hotspots mapped previously.

SPECIFIC COMMENTS
In the abstract the authors state that "We proposed the first computational approach, SSBlazer" for SSB detection. However a lot of the discussion is on a comparison to SSBERT, already developed by the authors, which appears to be a computational approach to SSB detection. The contribution of this paper needs to be clarified. Is it the software or the lightweight model?

I found the description of the algorithm and general approach could have been placed as the first subsection in Results perhaps under heading "Development of a new ML model for SSB detection" or similar. Since this is a novel approach and ML model it counts as a result. it would improve the readability of the manuscript as right now it skips directly to the details and is difficult to follow.

Fig 2b: Authors claim "Figure 2 b shows that SSBlazer significantly outperforms the other baseline machine learning models in both AU-ROC and AUPRC". As far as I can see there is no description of the default MLP and CNN models. This is also a bit of a "straw man" comparison since the default CNN could be chosen to be as simple as possible. Please provide details and how these were optimised for task at hand.

BERT/DNABERT/SSBERT was not adequately described in the text. (Might be alleviated if Methods:Model framework is moved into Results)

In the species evolutionary hierarchy analysis it appears that only chromosome 1 was used (if I understand correctly). Why is this? What limitations might this impose. This is not included in the discussion.

**Reviewer 2**

In this manuscript, Xu et al describe a new bioinformatics pipeline to predict DNA single-strand breaks (SSBs) from genomic sequences. They design a deep learning approach and use two data sets of genome-wide SSB patterns as a training set. Their algorithm takes into account peaks of SSBs (determined by a common peak calling algorithm) and their surrounding sequences (75 nt on either side). In this manner, they show that the algorithm is capable of predicting the (known) SSB pattern of a mouse cell line with good quality. Their analysis shows that CpG sequences are correlated with SSBs, consistent with an accumulation of breaks in gene regulatory elements. Finally, the authors apply their prediction algorithm to other vertebrate species and report a correlation between SSB patterns and evolutionary relationship.
This reviewer is not a computational biologist or bioinformatician and can therefore not competently evaluate the computational aspects of this work. I will therefore give my comments from a largely biological perspective.
Overall, I found the manuscript very difficult to read and understand. This is in parts due to my lack of expertise, in parts also to the use of terms by the authors that are not defined and therefore incomprehensible to the non-expert (e.g. AUROC, AUPRC), and sometimes also to poor English. Leaving these difficulties aside, I appreciate the authors' efforts in generating a tool for the quantitative, comparative analysis of DNA lesions. In contrast to mutation signatures, which are routinely collected and are now an important part of cancer research, tools to generate DNA lesion signatures are lacking, although they could undoubtedly reveal plenty of interesting and insightful information about DNA repair processes and genome instability. That said, the authors don't emphasize this very useful aspect of their research. Instead, they 'sell' their algorithm as a tool for predicting SSBs in different species. In my opinion, this is not the application that biologists would find most useful. The reason is that the authors' predictions assume that the biological mechanisms underlying the generation of SSBs are constant in all the species that they analyze and compare. For a comparison between human and mouse cell lines under undisturbed conditions, this may work quite well, but for more distantly related species it would be difficult. As an extreme example, budding yeast doesn't have CpG methylation and is therefore not expected to show a correlation between SSB patterns and CpG sequences. Likewise, the algorithm wouldn't work for the analysis of mutant cells with defects in specific repair pathways or under conditions of genotoxic stress where SSBs are generated by mechanisms not comparable to the spontaneous patterns that their predictions are based on. Thus, overall, I am wondering what the use of a prediction tool would be that can only be used for relatively closely related species under undisturbed conditions. Any conclusions from such predictions would likely have to be verified by sequencing after all.
As an experimental biologist, I am wondering, however, whether this tool could be used to tease out patterns of lesions or correlations between damage and sequences in a more general sense, i.e. for lesions other than just SSBs and different metabolic or cell cycle stages. Would it be possible to use the same tool for datasets that describe the genome-wide distribution of abasic sites or oxidative damage? In my view, it would be extremely interesting to try and generate signatures for these types of lesions and/or other cell states and then compare the outcome with other genomic features, in a manner analogous to what the authors did to reveal the correlation of SSBs with CpG sequences. Such signatures could also be used as biomarkers, for example, to identify what type(s) of DNA damage/process(es) could be mis-regulated in an experimental sample of interest. I would like to encourage the authors to work more closely with biologists and consider such ideas to realize the full potential of their method. As it stands now, I am not sure whether the tool would be of much use to the community of biologists.

Some specific comments on the text:

- Page 1 Line 61 - The authors claim that "The importance of SSBs has been underestimated since the dedicated cellular pathways that deal with every procedure of restoring SSBs, including SSB detection and repair procedures, have not been fully illustrated." I would argue that SSB processing, although maybe not fully understood, has been productively investigated for decades, and it is clear that it plays important roles. For example, mutations in proteins involved in SSB repair have obvious consequences on cellular fitness and human disease.

- The authors rely on MACS2 to identify "true" SSBs from their SSB-seq/SSingle datasets. They should make this clear in the main text of the manuscript. In our experience, due to the rather pervasive low-level distribution of SSBs throughout the genome, trying to find statistically significant hits with MACS2 can be tricky and potentially introduce biases. This limitation should be discussed. This issue also raises the question as to why, for training with the imbalanced datasets, the authors used sequences "randomly sampled from reference genomes" rather than using those positions not marked by MACS2 as "true" SSBs. These could represent more biologically/technically representative background than a randomly chosen sequence, and therefore improve the model?

- Figure 4 - The authors should clarify why they sampled sequences only from Chromosome 1.

- Figure 3 a-d - The authors should indicate which dataset was used in panels a-d at the top of the panel itself and/or in the legend, if it's not already included.

- Figure 3 a, c - The authors should indicate what each of the three graphs in panels a and c represents in the panel itself/legend/main text. Do they represent the sequence predictions produced from each one of the imbalanced datasets?

- Figures 3 b, d and 4 b: could the authors not simply be observing multicollinearity between SSB counts, CpG content and evolutionary hierarchy? In other words, how does CpG content relate to evolutionary hierarchy? For example, evolutionary hierarchy could define CpG content (is this, in fact, known?). Since CpG content is directly related to SSB count, probably through C methylation (as the authors point out), then evolutionary hierarchy must obviously also be, even though one does not necessarily directly impinge on the other. This potential limitation should be discussed.

- The manuscript should be proof-read by a native English speaker to eliminate various mistakes and poor expressions.

**Reviewer 3**

In this manuscript, the authors proposed SSBlazer, an explainable and scalable deep learning framework for genome-wide nucleotide-resolution SSB site prediction. I have the following issues that need the authors to address.

For datasets, the authors set the sample sequences to 151 bp long. They should explain why they chose the length setting. How the sequence length impacts the predictive performance? for example, long-distance information along the sequences. Moreover, the negative set construction is not correct. To be specific, they randomly sampled the negative sequences from genome. It usually brings bias for model training and evaluating. They should sample those non-SSB sites but quite similar with SSB sites (i.e. sharing similar patterns?) as negatives. Moreover, the sequence identity between positives and negatives is also a key factor that might generate the evaluation bias and impact the generalization ability.

As for a software paper, the authors refer not much about the software. The presented webserver is quite simple. Not much information is given, but only prediction results only. It would be more practical if the method could be applied to whole-genome prediction.

As for the proposed method, the novelty is relatively low in algorithms. They applied existing commonly used deep learning models to implement their methods. Moreover, the performance of the proposed models is quite important. As for the predictive performance, it is suggested to compare the proposed methods with more advanced deep learning architectures. Moreover, they should test their proposed methods on the genome-wide annotation.

As for the imbalanced setting, I doubt the authors' settings could simulate the real SSB distributions. They should provide more evidence. Moreover, they should discuss more regarding this issue.

The writing of the manuscript should be polished further.

**Authors’ response to reviewers**

Reviewer #1

The manuscript by Xu and colleagues describes a new method to map genomic sites prone to single strand breaks using a flexible machine learning approach. This method is applied to the genomes of 216 vertebrates that have not had SSB hotspots mapped previously.

We thank the reviewer for providing a concise summary of our manuscript. This summary effectively captures the essence of the research, highlighting the development of a novel method for mapping genomic sites prone to single-strand breaks (SSBs).

SPECIFIC COMMENTS:
1. In the abstract the authors state that "We proposed the first computational approach, SSBlazer" for SSB detection. However a lot of the discussion is on a comparison to SSBERT, already developed by the authors, which appears to be a computational approach to SSB detection. The contribution of this paper needs to be clarified. Is it the software or the lightweight model?

Thank you for your thoughtful feedback. We understand the confusion in our initial presentation, and we agree that the contribution of this paper needs to be clarified. The primary contribution of this paper is the introduction of SSBlazer, a novel, lightweight model for SSB detection.

SSBERT also developed by us, is a more resource-intensive model, and while it does address a similar problem, the focus of this paper is to introduce a model that achieves comparable performance with significantly less computational requirements. To avoid any ambiguity, we have renamed the SSBERT into SSBlazer-LM (the language model version of SSBlazer) and relocated both the figure and the textual description of SSBlazer to the Supplementary in the revised manuscript. Following your advice, we have removed SSBlazer-LM in Figure 1 and renamed all representees of SSBERT to SSBlazer-LM.

We have moved the description of SSBlazer-LM is moved to Supplementary: Baseline Model:
“SSBlazer-LM. This model is a BERT-based language model, and all the parameters are initialized with those of DNABERT [1], which is a pre-trained language model on the human reference genome (GRCh38.p13). SSBlazer-LM uses k-mer representations of the DNA sequence as input tokens, where tokens are first generated from the input sequence and then converted into numeral IDs by a predetermined mapping. In this stage, token IDs are used to obtain token embeddings. Segment embeddings and position embeddings are determined by the sequence and the position of each nucleotide. All these embeddings are vectors with a length of 768, and these vectors are then fed into an element-wise addition module to generate the final embeddings of the input sequence. Then, these embedding vectors are processed by 12 transformer blocks to obtain the final context feature, and each transformer block consists of 12 attention heads and 768 hidden units. The final embedding output (E[CLS]) of the last hidden layer is regarded as the sequence feature and will be fed into the classification layer for prediction.”

The contribution of this paper is a novel software for SSB prediction. We experimented two versions of the model, SSBlazer and SSBlazer-LM. Intriguingly, SSBlazer, despite having substantially fewer parameters, was found to deliver performance on par with SSBlazer-LM. Due to its lightweight design, SSBlazer is capable of facilitating large-scale applications, and was therefore selected as the final model for our SSB prediction software.

To delineate our contributions more clearly, we have added the Overview of SSBlazer section in Results:
“SSBlazer is designed to predict SSB tendencies using local genome windows. This method combines deep learning, activation propagation, and residual and attention strategies to first predict the SSB sites, then quantify the contribution of the break site genomic context, and finally identify the genomic pattern of SSBs, such as the TGCC motif and a high frequency of CpG. As illustrated in Figure 1a, the sequencing data of S1 END-seq and SSiNGLe-ILM undergo a standard pipeline including quality control, genome alignment, peak calling and SSB site determination to collect the genuine SSB sites in a genome-wide manner [1]. To construct the sequence-based model, we extracted the sequence context of the determined SSB sites as input. During the model prediction (Figure 1b), the sequences are involved in two-stream feature extraction. The full-length sequence goes through the residual block and the self-attention block for detecting interaction within the sequence, while the center 9 bp goes through the inception block, given the central part of SSB shares higher importance, and then the self-attention block, to capture useful information from the raw sequence. Finally, these two-stream extracted feature vectors are concatenated and fed into an MLP for SSB tendency prediction. In addition, there is further interest in identifying and quantifying the genomic context for SSB sites (Figure 1c). A previous study [2] extracted the motif via the activation of the first layer in the deep-learning model, but this approach overlooks the complexity of the downstream layers and struggles to quantify the motif’s contribution. To address this, an activation backpropagation method (DeepLIFT [3]) was employed. The prediction of a specific sequence is backpropagated through the entire network back to the original inputs to assign contribution scores to the motifs. Importantly, this process considers the entire deep-learning model and computes the score in a purely data-driven manner. Based on interpretation analysis, it was discovered that SSBlazer captures the motif TGCC in the central region and a high frequency in the flanking region. Additionally, the model was interpolated on all known vertebrate genomes, depicting the genome-wide SSB site landscapes on vertebrate genomes. SSBlazer incorporates functionality for mutation analysis to profile alterations in the SSB landscape, identifying SSB-related SNPs.”

2. I found the description of the algorithm and general approach could have been placed as the first subsection in Results perhaps under heading "Development of a new ML model for SSB detection" or similar. Since this is a novel approach and ML model it counts as a result. it would improve the readability of the manuscript as right now it skips directly to the details and is difficult to follow.

We agree that the presentation of the development of our novel machine learning model could be better placed as a result, given its significance in our study. We also appreciate your feedback on the readability of the manuscript and understand how the current organization may be challenging to follow.

Following your suggestion, we've added a new Overview of SSBlazer subsection to the Results in our revised manuscript.
3. Fig 2b: Authors claim "Figure 2 b shows that SSBlazer significantly outperforms the other baseline machine learning models in both AU-ROC and AUPRC". As far as I can see there is no description of the default MLP and CNN models. This is also a bit of a "straw man" comparison since the default CNN could be chosen to be as simple as possible. Please provide details and how these were optimised for task at hand.

We are grateful for your meticulous feedback and concur that it would indeed be beneficial to provide more comprehensive information about the default MLP and CNN models utilized in our study, to ensure a fair comparison.

We have therefore added an in-depth examination of both the MLP and CNN models, which includes a review of diverse model architectures. The hyperparameters used are identical to those of SSBlazer, featuring a learning rate of 0.001, implemented with a linear warm-up strategy, a batch size of 2048, and training duration spanning 50 epochs.

Conv Layers Channels Kernel Size AUROC AUPRC
2 [32, 32] [3, 3] 0.9348 0.9300
3 [32, 32, 64]
[3, 3, 3] 0.9459 0.9425
4 [32, 32, 64,64]
[3, 3, 3, 3] 0.9532 0.9508
2 [64,64]
[3, 3] 0.9367 0.9319
3 [64,64,128] [3, 3, 3] 0.9503 0.9471
4 [64,64,128,128] [3, 3, 3, 3] 0.9548 0.9530
Table R1: Ablation for baseline CNN models

Hidden Layers Hidden Dimension AUROC AUPRC
1 1024 0.7808 0.7640
2 1024 0.7711 0.7599
3 1024 0.7723 0.7541
4 1024 0.7657 0.7453
5 1024 0.7655 0.7434
Table R2: Ablation for baseline MLP models

We appreciate your suggestions, and have added the description of the default MLP and CNN models in Supplementary: Baseline model:

“The MLP model uses a flattened vector of the one-hot encoded sequence as its input. From the ablation study, we have selected the MLP model with one hidden layer and a hidden dimension of 1024 as our baseline model, as it achieved the highest AUROC and AUPRC scores of 0.7808 and 0.7640 respectively.

The input for the CNN model is identical to the SSBlazer, which is a 251*4 matrix. It is important to note that SSBlazer is also a CNN-based model. Therefore, we evaluated several fundamental CNN models as baselines for comparison. From our evaluations, the best-performing CNN model consisted of four convolutional layers, with the numbers of channels being [64, 64, 128, 128] and the kernel sizes being [3, 3, 3, 3]. This model yielded the highest AUROC and AUPRC scores of 0.9548 and 0.9530 respectively, and it has been chosen as our baseline CNN model.”

3. BERT/DNABERT/SSBERT was not adequately described in the text. (Might be alleviated if Methods:Model framework is moved into Results)

We appreciate your feedback on the need for a more detailed account of SSBlazer-LM (SSBERT). In response, we have relocated the description of SSBlazer-LM to the Supplementary: Baseline model subsection to enhance comprehension of our approach.

4. In the species evolutionary hierarchy analysis it appears that only chromosome 1 was used (if I understand correctly). Why is this? What limitations might this impose. This is not included in the discussion.

We appreciate the reviewer's question regarding the species evolutionary hierarchy analysis and the use of only chromosome 1. The limited focus on Chromosome 1 was indeed a specific choice in our analysis, and we recognize the importance of explaining this choice and discussing its implications.

The choice to focus on Chromosome 1 in the species evolutionary hierarchy analysis was made to provide a simplified and illustrative example of how our method could be applied to assess correlations with evolutionary relationships. Chromosome 1 was selected as a representative region to showcase the concept, as it allowed for a clear demonstration without the complexities associated with analyzing the entire genome.
However, we acknowledge that this choice may have limitations, particularly in terms of capturing the full complexity of genome-wide correlations with evolutionary relationships. To address this concern, we revised the manuscript to explicitly state the rationale behind this choice and discuss its limitations in the context of the analysis. Additionally, we emphasized that the approach can be extended to include multiple chromosomes.

Additionally, we pursued further research on the conditions of different chromosomes and their relationship with the whole genome. We conducted simultaneous analysis on chromosomes 1, 2, and 3 (Figure R4.) and the results revealed similar trends (Chr1: r=0.86, p=2.9e-64; Chr2: r=0.87, p=1.6e-62; Chr3: r=0.84, p=7.9e-38). The correlation pattern obtained from whole-genome sampling was slightly different (Full Genome: r=0.52, p=3.1e-16). We speculate that during the assembly process, the genomes of these insufficiently studied species contained some low-quality or unrecognizable chromosome data, which prevented the model from accurately identifying the SSB breakage frequency. Concurrently, the evaluation strategy of leave-chromosome1-out has been widely implemented[2, 3].


Figure R1: Genome-wide correlation analysis demonstrated the association between CpG content and predicted SSB counts with different sampling methods.
 
Reviewer #2

In this manuscript, Xu et al describe a new bioinformatics pipeline to predict DNA single-strand breaks (SSBs) from genomic sequences. They design a deep learning approach and use two data sets of genome-wide SSB patterns as a training set. Their algorithm takes into account peaks of SSBs (determined by a common peak calling algorithm) and their surrounding sequences (75 nt on either side). In this manner, they show that the algorithm is capable of predicting the (known) SSB pattern of a mouse cell line with good quality. Their analysis shows that CpG sequences are correlated with SSBs, consistent with an accumulation of breaks in gene regulatory elements. Finally, the authors apply their prediction algorithm to other vertebrate species and report a correlation between SSB patterns and evolutionary relationship.
This reviewer is not a computational biologist or bioinformatician and can therefore not competently evaluate the computational aspects of this work. I will therefore give my comments from a largely biological perspective.

We thank the reviewer for providing a biological perspective on our manuscript. Your comments offer valuable insights into the research described in the paper. It's important to have perspectives from both computational and biological experts to comprehensively evaluate our work.

Comments/Questions/Suggestions:
1. Overall, I found the manuscript very difficult to read and understand. This is in parts due to my lack of expertise, in parts also to the use of terms by the authors that are not defined and therefore incomprehensible to the non-expert (e.g. AUROC, AUPRC), and sometimes also to poor English.

We appreciate your feedback on our manuscript. We apologize for any difficulty you encountered while reading and understanding our work. We have taken your comments seriously and made every effort to improve the clarity and accessibility of our manuscript.

We understand that the topic of our manuscript may not be familiar to all readers. To address this, we revised the Introduction section to provide a more comprehensive overview of the subject matter, making it more accessible to a broader audience. We have added an Overview of SSBlazer subsection in Results:
“SSBlazer is designed to predict SSB tendencies using local genome windows. This method combines deep learning, activation propagation, and residual and attention strategies to first predict the SSB sites, then quantify the contribution of the break site genomic context, and finally identify the genomic pattern of SSBs, such as the TGCC motif and a high frequency of CpG. As illustrated in Figure 1a, the sequencing data of S1 END-seq and SSiNGLe-ILM undergo a standard pipeline including quality control, genome alignment, peak calling and SSB site determination to collect the genuine SSB sites in a genome-wide manner [1]. To construct the sequence-based model, we extracted the sequence context of the determined SSB sites as input. During the model prediction (Figure 1b), the sequences are involved in two-stream feature extraction. The full-length sequence goes through the residual block and the self-attention block for detecting interaction within the sequence, while the center 9 bp goes through the inception block, given the central part of SSB shares higher importance, and then the self-attention block, to capture useful information from the raw sequence. Finally, these two-stream extracted feature vectors are concatenated and fed into an MLP for SSB tendency prediction. In addition, there is further interest in identifying and quantifying the genomic context for SSB sites (Figure 1c). A previous study [2] extracted the motif via the activation of the first layer in the deep-learning model, but this approach overlooks the complexity of the downstream layers and struggles to quantify the motif’s contribution. To address this, an activation backpropagation method (DeepLIFT [3]) was employed. The prediction of a specific sequence is backpropagated through the entire network back to the original inputs to assign contribution scores to the motifs. Importantly, this process considers the entire deep-learning model and computes the score in a purely data-driven manner. Based on interpretation analysis, it was discovered that SSBlazer captures the motif TGCC in the central region and a high frequency in the flanking region. Additionally, the model was interpolated on all known vertebrate genomes, depicting the genome-wide SSB site landscapes on vertebrate genomes. SSBlazer incorporates functionality for mutation analysis to profile alterations in the SSB landscape, identifying SSB-related SNPs.”

We acknowledge that we used acronyms and technical terms without proper explanation (AUROC, AUPRC). We have now added a subsection titled Evaluation metrics under the Methods section:
“In this study, we have chosen AUROC and AUPRC as our evaluation metrics due to their effectiveness in measuring the performance of classification models:


Both of these metrics provide an aggregate measure of performance across all possible classification thresholds. The AUROC is particularly useful as it is invariant to the class distribution or the threshold setting. The AUROC ranges from 0 to 1, with a value of 0.5 indicating a random model and a value of 1 indicating a perfect model. The higher the AUROC, the better the model's performance at discriminating between the positive and negative classes. AUPRC is based on precision and recall. Precision measures the proportion of true positive predictions among all positive predictions, while recall measures the proportion of true positive predictions among all true positive instances. AUPRC is especially useful when the cost of false positives and false negatives is different, as it allows for the identification of the optimal classification threshold that minimizes the cost.

We apologize for any language issues in the manuscript. We have thoroughly reviewed and edited the manuscript for language and grammar to ensure it meets the highest standards of clarity and coherence.

2. Leaving these difficulties aside, I appreciate the authors' efforts in generating a tool for the quantitative, comparative analysis of DNA lesions. In contrast to mutation signatures, which are routinely collected and are now an important part of cancer research, tools to generate DNA lesion signatures are lacking, although they could undoubtedly reveal plenty of interesting and insightful information about DNA repair processes and genome instability. That said, the authors don't emphasize this very useful aspect of their research. Instead, they 'sell' their algorithm as a tool for predicting SSBs in different species. In my opinion, this is not the application that biologists would find most useful. The reason is that the authors' predictions assume that the biological mechanisms underlying the generation of SSBs are constant in all the species that they analyze and compare. For a comparison between human and mouse cell lines under undisturbed conditions, this may work quite well, but for more distantly related species it would be difficult. As an extreme example, budding yeast doesn't have CpG methylation and is therefore not expected to show a correlation between SSB patterns and CpG sequences. Likewise, the algorithm wouldn't work for the analysis of mutant cells with defects in specific repair pathways or under conditions of genotoxic stress where SSBs are generated by mechanisms not comparable to the spontaneous patterns that their predictions are based on. Thus, overall, I am wondering what the use of a prediction tool would be that can only be used for relatively closely related species under undisturbed conditions. Any conclusions from such predictions would likely have to be verified by sequencing after all.

We appreciate your thoughtful comments and the recognition of our efforts to develop a tool for the quantitative analysis of DNA lesions. Your insights are valuable, and we addressed your concerns in our revised manuscript. Here are our responses to your specific points:

(1) We understand your concern regarding the emphasis on predicting SSBs in different species and the potential limitations of this application. In the revised manuscript, we indeed highlight the broader utility of our tool in exploring DNA repair processes and genome instability. We provide additional context in and examples to demonstrate how our tool can be applied beyond species comparisons under undisturbed conditions. We concur that the hierarchical analysis could garner interest from biologists. Furthermore, in the Web Server section, we have included an analysis of pathogenic SNPs, adding another layer of depth to the potential applications of our tool:
“The current version of SSBlazer trained on the S1 END-seq dataset and the SSiNGLe-ILM dataset while SSBlazer is a portable and well-organized model, which can smoothly transition to the unified dataset and other available SSB detection datasets (e.g., SSB-seq [4], GLOE-seq [5]). We provided a retraining tutorial in https://sxu99.gitbook.io/ssblazer/use-cases/train-a-new-model. This adaptability can substantially improve SSBlazer’s generalization ability, making it a valuable tool in the continued exploration and understanding of the SSBreakome.”


“In this study, we have depicted vast landscapes of SSB sites for diverse species via SSB frequency using SSBlazer, but there are still many unexplored SSB-related applications for this tool. For instance, the SSB landscape within a target gene can be used to evaluate gene stability. To illustrate, we conducted a SNP comparison analysis on Alzheimer’s Disease (AD) patients. We collected pathogenic SNP data from ClinVar [26] and applied SSBlazer to assess how such genomic substitutions might influence the local SSB landscape. Our findings (Figure 4 d) indicate that a particular SNP (rs122356) in ClinVar alters the SSB landscape against the reference genome, suggesting that pathogenic SNPs may promote the occurrence of AD via SSB-related pathways.”

The pathogenic SNPs analysis is also included in Figure 1c and Figure 4c.

(2) We appreciate your insight regarding our model's assumption of constant biological mechanisms underlying SSB generation across species. In the revised manuscript, we have clarified this limitation and have provided a nuanced discussion about our tool's applicability, including potential constraints when applied to species with unique repair mechanisms, mutants, or under genotoxic stress conditions.

Our model was established using datasets from mice and humans, and the model's generalizability was evaluated through cross-dataset methods. We completely agree with your point that this model may not be applicable to organisms like yeast that do not undergo CpG methylation modification. Consequently, we have focused our large-scale analyses on vertebrates, which do exhibit CpG methylation modification.

In accordance with the editor's suggestion,, we have reformatted this manuscript into a software format. In this context, our model serves a use case and reveals some intriguing biological phenomena. However, these interesting findings require further exploration and validation by biologists. And we have also addressed the potential biases arising from varying genome sizes by employing normalized sampling.

These changes could be found in the subsextion SSBlazer explores SSB-related applications: Case study:
“The successful construction of SSBlazer has enabled a plethora of applications related to SSBs. SSB frequency is considered an implicit feature of genomic integrity [6], which could be linked to differences in the evolutionary process across diverse species. Cross-species evaluation in human and mouse datasets indicated that SSBs across diverse species might share similar genomic patterns. SSBlazer accurately captures these patterns, demonstrating robust cross-species generalization ability. Thus, we first collected all the available vertebrate reference genomes (212 non- redundant species and included Actinopteri, Mammalia, Reptillia, Lepidosauria, Chondrichthyes, Amphibia, Aves.) from the Genbank [7] and RefSeq [8]. Given the biases that might arise due to varying genome sizes, we employed normalized sampling. Specifically, we generated 1 million sample sequences, each with 251 bp, from chromosome 1 of each reference genome [3]. Finally, we applied SSBlazer to this large collection of sequences to identify putative SSB sites. This allowed us to estimate the SSB frequency and, consequently, the genome integrity of each species. Remarkly, the frequency of SSB is highly variable among different species, ranging from 7,600 in thirteen-lined ground squirrel (Ictidomys tridecemlineatus) to 130,088 in Alaskan stickleback (Gasterosteus aculeatus) and a clear trend along the evolutionary process is observed. The phylogenetic diagram (Figure 5 a) and the violin plot (Figure 5 b) indicate the SSB count distribution at the species and class level. These results reveal that Actinopteri and Chondrichthyes had relatively high levels of SSB frequency, while Reptilia, Mammalia and Aves share fewer SSB frequencies. This broad analysis provides a comprehensive view of SSB frequency across a diverse range of vertebrates, shedding light on its role in genomic integrity and evolutionary processes.”

(3) Given the available collections of mice and human dataset, we have demonstrated the model's generalizability to the best of our ability. In our case study, we focused our investigation on vertebrates. Admittedly, we require more experimental validation to substantiate the hypotheses generated by the model. We believe that, in the future, the inclusion of data from a greater variety of species will effectively enhance our model's generalizability. Concurrently, we provide a portable model, as well as a tutorial for retraining (https://sxu99.gitbook.io/ssblazer/use-cases/train-a-new-model). We acknowledge that predictions should ideally be verified by experimental validation through sequencing. We emphasize in the manuscript that our tool serves as a hypothesis-generating and exploratory tool, which can guide experimentalists in selecting regions of interest for further study and validation.

3. As an experimental biologist, I am wondering, however, whether this tool could be used to tease out patterns of lesions or correlations between damage and sequences in a more general sense, i.e. for lesions other than just SSBs and different metabolic or cell cycle stages. Would it be possible to use the same tool for datasets that describe the genome-wide distribution of abasic sites or oxidative damage? In my view, it would be extremely interesting to try and generate signatures for these types of lesions and/or other cell states and then compare the outcome with other genomic features, in a manner analogous to what the authors did to reveal the correlation of SSBs with CpG sequences. Such signatures could also be used as biomarkers, for example, to identify what type(s) of DNA damage/process(es) could be mis-regulated in an experimental sample of interest. I would like to encourage the authors to work more closely with biologists and consider such ideas to realize the full potential of their method. As it stands now, I am not sure whether the tool would be of much use to the community of biologists.

We appreciate your feedback and your perspective as an experimental biologist. Your suggestions for expanding the utility of our tool to encompass a broader range of DNA lesions and cell states are insightful.

(1) We completely agree with your suggestion that our tool could be extended to analyze patterns of lesions beyond just SSBs and consider different metabolic or cell cycle stages. In fact, the methodology underlying our tool is versatile and can be adapted to various types of DNA damage. However, the absence of the different subtype of DNA lesions lead to incapability of DNA lesions subtype prediction. We highlighted the flexibility of our approach and its potential for analyzing diverse types of DNA lesions in Discussion:
“The current version of SSBlazer trained on the S1 END-seq dataset and the SSiNGLe-ILM dataset while SSBlazer is a portable and well-organized model, which can smoothly transition to the unified dataset and other available SSB detection datasets (e.g., SSB-seq [4], GLOE-seq [5]). We provided a retraining tutorial in https://sxu99.gitbook.io/ssblazer/use-cases/train-a-new-model. This adaptability can substantially improve SSBlazer’s generalization ability, making it a valuable tool in the continued exploration and understanding of the SSBreakome.”

(2) Your idea of generating signatures for various types of DNA lesions and cell states and comparing them with genomic features is compelling. Such comparisons could indeed reveal valuable insights into DNA damage and repair processes. We have incorporated this concept and integrated the genome-wide SSB site landscape in the webserver, which typically intuitive and useful for the scientific researchers.

(3) We wholeheartedly embrace your suggestion to collaborate more closely with biologists. We believe that interdisciplinary collaboration can help bridge the gap between computational tools and experimental research. In the revised manuscript, we have emphasized the importance of such collaborations and encouraged researchers to explore the potential applications of our tool within their specific fields of interest.
“SSBlazer elucidates the conjectural genomic landscapes of single-strand breaks across all accessible vertebrate genomes in the cross-species analysis. Remarkably, the prospective SSB genomic landscapes derived from a study of 212 vertebrates demonstrate a potential hypothesis about the correlation between SSB frequency and the respective evolutionary hierarchy, which needs to be further experimentally validated.”

“SSBlazer offers an easily navigable web server, primed to tackle numerous yet-to-be-explored issues related to single-strand breaks, such as the analysis of pathogenic SNP mutations. In addition, the server includes ready-to-use SSB landscapes for 212 vertebrate genomes, visualized with the Integrative Genomics Viewer, thereby broadening its utility for a variety of applications.”

(4) We can re-train SSBlazer to adopt a various types of DNA damage. For instance, we collected double-strand break (DSB) sites from GSM4047457, which were identified by BLISS-seq of MCF7 cell line, and constructed training and testing datasets following a similar process as for SSBs. The model, re-trained on the DSB dataset, showed satisfactory performance (AUROC=0.8277, AUPRC=0.7788), indicating that SSBlazer can adapt to different damage types with re-training. We have provided a detailed manual for training a new model at https://sxu99.gitbook.io/ssblazer/use-cases/train-a-new-model:

Figure R2: Screenshot of Train a New Model part in the online manual.

Please note, however, that training SSBlazer from scratch could require substantial computational resources. We understand the challenges this might present and, in an effort to foster collaborative research, we would be more than happy to assist in model training should researchers be willing to share their datasets with us.


Specific comments on the text:
1. Page 1 Line 61 - The authors claim that "The importance of SSBs has been underestimated since the dedicated cellular pathways that deal with every procedure of restoring SSBs, including SSB detection and repair procedures, have not been fully illustrated." I would argue that SSB processing, although maybe not fully understood, has been productively investigated for decades, and it is clear that it plays important roles. For example, mutations in proteins involved in SSB repair have obvious consequences on cellular fitness and human disease.

We appreciate your comment and the opportunity to clarify our statement regarding the importance of SSBs.

We appreciate you highlighting that SSB processing has been productively investigated, and the significance of SSBs in cellular processes and human diseases is well-established. We did not intend to downplay the importance of SSBs but rather to emphasize that despite the existing research, there may still be aspects of SSB detection and repair pathways that are not fully elucidated. We acknowledge the extensive research on SSBs, their roles in cellular fitness, and their relevance to human diseases while also acknowledging the potential for further discoveries in the field.

We have revised the statement on page 1, line 61, to reflect this clarification:
“Although the importance of SSBs in cellular processes and human diseases is well-established, the specific cellular pathways that manage each step of SSB restoration, encompassing both SSB detection and repair procedures, have not yet been fully elucidated. Typically, defects in these pathways can lead to genotoxic stress, embryonic lethality, and various neurodegenerative diseases. Consequently, outlining the SSB landscape could offer fresh insights into these disease mechanisms and lay the groundwork for corresponding therapeutic approaches.”

2. - The authors rely on MACS2 to identify "true" SSBs from their SSB-seq/SSingle datasets. They should make this clear in the main text of the manuscript. In our experience, due to the rather pervasive low-level distribution of SSBs throughout the genome, trying to find statistically significant hits with MACS2 can be tricky and potentially introduce biases. This limitation should be discussed. This issue also raises the question as to why, for training with the imbalanced datasets, the authors used sequences "randomly sampled from reference genomes" rather than using those positions not marked by MACS2 as "true" SSBs. These could represent more biologically/technically representative background than a randomly chosen sequence, and therefore improve the model?

Thank you for your valuable comments regarding the use of MACS2 for identifying SSBs in our S1 END-seq/SSiNGLe datasets and the approach we used for training with imbalanced datasets. Your concerns and suggestions are well-founded, and we appreciate the opportunity to address them. Here's our response:

1) We acknowledge the importance of clearly stating in the manuscript that we utilized MACS2 for identifying "true" SSBs from our datasets. We would like to clarify that our choice to use MACS2 for identifying "true" SSBs is grounded in the procedure outlined in the original paper [9, 10] that we collected the raw data. We included a specific mention of this in the Methods section to provide transparency and clarity to the readers.
“The S1 END-seq data of human i3Neurons cell line was collected from the study of Wu et al. [18]. This data was processed using the standard bioinformatics pipeline for END-seq [37]. The initial step involved downloading the original sequencing data from the Gene Expression Omnibus (GEO) database (GSE167259) and collecting the raw reads of the ddN S1 END-seq sample via the SRA Run Selector. Quality control was subsequently conducted using fastqc (v.0.11.9), and trim galore (v.0.6.4) was employed to remove adapters and low-quality reads. The clean reads of the END-seq were then aligned to the Homo sapiens genome (hg19) using bowtie (v.1.1.2), and a sorted bam file was created with the aid of samtools (v.1.14). After genome alignment, peak calling was performed with MACS2 (v.2.2.7.1), and the peak summits were gathered to generate SSB sites (positive sample). Given the summit coordinate, we extracted the 125 bp sequence context upstream and downstream from the Homo sapiens genome (hg19) to enhance performance, resulting in a final input sequence length of 251 bp. Furthermore, to augment the data, we introduced a reverse complementary sequence for each positive sample, as S1 END-seq is a non-strand-specific SSB detection approach.”

2) We appreciate the opportunity to clarify the process for generating negative (background) sequences in our imbalanced datasets. In contrast to a simple random sampling from the entire reference genomes, we specifically sampled from positions not identified as "true" SSBs by MACS2. This approach aligns with your suggestion. We apologize if this was not made clear in our initial manuscript.

3) We followed the method used in DeepBind [11] where dinucleotide-shuffled sequences were generated as negative samples. This method preserves the count of all 16 dinucleotides (AA, AC, ..., GT, TT) from the original sequence, thereby maintaining a relatively high sequence similarity. However, we found that the dinucleotide-shuffle generated samples did not perform better in distinguishing SSB sites from background on the genome compared to the dataset constructed with randomly sampled negative samples. In fact, the AUROC decreased from 0. 9626 to 0. 8170 (Figure R3).

Figure R3: Receiver operating characteristic (ROC) curve comparing the performance of two models based on different negative sampling methods.

We have provided a detailed description of the negative sequence sampling process in the SSBlazer accurately predicts SSB sites subsection:
“The dinucleotide sampling is included in the Supplementary: Evaluating the efficacy of dinucleotide-shuffled sequences in distinguishing SSB sites, and we found that the
model trained on such a dataset fails to capture the genomic occurrence pattern of
SSBs.”

3. - Figure 4 - The authors should clarify why they sampled sequences only from Chromosome 1.

We appreciate the reviewer's question regarding Figure 4 and the specific choice to sample sequences from Chromosome 1. The selection of Chromosome 1 for sampling was not explained adequately in the manuscript, and we understand the need to provide clarity on this matter.

The choice to focus on Chromosome 1 in the species evolutionary hierarchy analysis was made to provide a simplified and illustrative example of how our method could be applied to assess correlations with evolutionary relationships. Chromosome 1 was selected as a representative region to showcase the concept, as it allowed for a clear demonstration without the complexities associated with analyzing the entire genome.
However, we acknowledge that this choice may have limitations, particularly in terms of capturing the full complexity of genome-wide correlations with evolutionary relationships. To address this concern, we revised the manuscript to explicitly state the rationale behind this choice and discuss its limitations in the context of the analysis. Additionally, we emphasized that the approach can be extended to include multiple chromosomes.

Additionally, we pursued further research on the conditions of different chromosomes and their relationship with the whole genome. We conducted simultaneous analysis on chromosomes 1, 2, and 3 (Figure R4.) and the results revealed similar trends (Chr1: r=0.86, p=2.9e-64; Chr2: r=0.87, p=1.6e-62; Chr3: r=0.84, p=7.9e-38). The correlation pattern obtained from whole-genome sampling was slightly different (Full Genome: r=0.52, p=3.1e-16). We speculate that during the assembly process, the genomes of these insufficiently studied species contained some low-quality or unrecognizable chromosome data, which prevented the model from accurately identifying the SSB breakage frequency. Concurrently, the evaluation strategy of leave-chromosome1-out has been widely implemented[2, 3].


Figure R4: Genome-wide correlation analysis demonstrated the association between CpG content and predicted SSB counts with different sampling methods.


4. - Figure 3 a-d - The authors should indicate which dataset was used in panels a-d at the top of the panel itself and/or in the legend, if it's not already included.

We apologize for any confusion caused by the layout of Figure 3 (now Figure 2). It seems you might be referring to Figure 2 e-g. We intended to use these three graphs to demonstrate that increasing the imbalance ratio in the training dataset can reduce the number of false positives. The heatmaps represent the prediction results for the true SSB sites on the genome and their surrounding 125 bp, not the prediction results for the imbalanced datasets.

5. - Figure 3 a, c - The authors should indicate what each of the three graphs in panels a and c represents in the panel itself/legend/main text. Do they represent the sequence predictions produced from each one of the imbalanced datasets?

We have added descriptive legends for each dataset. These three sequences are marked as '1' (label=1 and prediction=1) in our constructed database. The purpose of this contribution score analysis is to investigate which bases assist the model in identifying the central position of the sequence as an SSB break point. The greater the contribution score, the greater the contribution that specific base makes to the model's determination of the sequence as a break point. This information could be found in the subsection: Model interpretation:
“To interpret the proposed model intuitively, we performed the integrated gradient analysis [23] to illustrate how the individual nucleic acid influences the prediction outcome and determine potential motifs of predicted SSB sites. In contrast to extracting motifs from the putative SSB site context sequences, the integrated gradient analysis is a comprehensive gradient-based axiomatic attribution method that computes a contribution score for each input feature compared to a baseline. It can efficiently visualize the genomic pattern that the model captures. In this study, integrated gradient analysis emphasizes the contribution score of each nucleotide in the sequence containing an SSB site.”

6. - Figures 3 b, d and 4 b: could the authors not simply be observing multicollinearity between SSB counts, CpG content and evolutionary hierarchy? In other words, how does CpG content relate to evolutionary hierarchy? For example, evolutionary hierarchy could define CpG content (is this, in fact, known?). Since CpG content is directly related to SSB count, probably through C methylation (as the authors point out), then evolutionary hierarchy must obviously also be, even though one does not necessarily directly impinge on the other. This potential limitation should be discussed.

Thank you for your insightful comment. You raise an important point about the potential multicollinearity between SSB counts, CpG content, and evolutionary hierarchy. This is indeed a complex relationship, and further investigation is needed to fully elucidate the specific interactions.

CpG content and its relationship to evolutionary hierarchy is a complex and intriguing area of study. While there is evidence to suggest that CpG content can be influenced by evolutionary processes, the exact mechanisms are not fully understood and can vary across different species and genomic regions.

Your point that the CpG content relates to the SSB count, likely through C methylation, is well taken. It is indeed likely that the evolutionarily driven variation in CpG content could indirectly influence SSB count, potentially confounding the relationship between SSB count and evolutionary hierarchy.

However, it's important to note that while these variables may be interconnected, it does not inherently negate the individual significance or the potential for independent effects of each factor. The potential for multicollinearity is an important consideration, and we appreciate that you've highlighted this aspect.

We agree that this potential limitation merits further discussion and will consider this in our future work. Following the editor's suggestion, we have reformatted this manuscript into a Software format. Here, our model offers a use case and reveals some intriguing biological phenomena including evolutionary hierarchy analysis. However, these interesting findings require further exploration and validation by biologists. Your feedback is instrumental in refining our understanding of these complex relationships and improving the robustness of our research.

7. - The manuscript should be proof-read by a native English speaker to eliminate various mistakes and poor expressions.

Thank you for your feedback regarding the language and grammar in the manuscript. We appreciate your input, and we agree that proofreading by a native English speaker is essential to improve the quality of the manuscript. 
Reviewer #3

In this manuscript, the authors proposed SSBlazer, an explainable and scalable deep learning framework for genome-wide nucleotide-resolution SSB site prediction. I have the following issues that need the authors to address.

1. For datasets, the authors set the sample sequences to 151 bp long. They should explain why they chose the length setting. we How the sequence length impacts the predictive performance? for example, long-distance information along the sequences. Moreover, the negative set construction is not correct. To be specific, they randomly sampled the negative sequences from genome. It usually brings bias for model training and evaluating. They should sample those non-SSB sites but quite similar with SSB sites (i.e. sharing similar patterns?) as negatives. Moreover, the sequence identity between positives and negatives is also a key factor that might generate the evaluation bias and impact the generalization ability.

We are grateful to the reviewer for pointing out that our previous method of constructing dataset might sound concerning. We agree that the sequence length and the construction of the negative set are important factors that could potentially impact the predictive performance and evaluation of our model.

Regarding the sequence length, we have tested various lengths ranging from 51 to 501 bp. We found that increasing the sequence length could indeed improve performance. However, considering both performance and computational cost, we finally opted for a length of 251 bp.


Figure R5: The AUROC and AUPRC value of different input sequence lengths.

As for the construction of the negative set, we understand your concerns about potential bias introduced by randomly sampling the negative sequences from the genome. To mitigate this, we followed the method used in DeepBind [11] where dinucleotide-shuffled sequences were generated as negative samples. This method preserves the count of all 16 dinucleotides (AA, AC, ..., GT, TT) from the original sequence, thereby maintaining a relatively high sequence similarity.

However, we found that the dinucleotide-shuffle generated samples did not perform better in distinguishing SSB sites from background on the genome compared to the dataset constructed with randomly sampled negative samples. In fact, the AUROC decreased from 0. 9626 to 0. 8170 (Figure R6).

Figure R6: Receiver operating characteristic (ROC) curve comparing the performance of two models based on different negative sampling methods.

We have provided a detailed description of the negative sequence sampling process in the SSBlazer accurately predicts SSB sites section:.

“The dinucleotide sampling is included in the Supplementary section 1.1. and the
model trained on such a dataset fails to capture the genomic occurrence pattern of
SSBs.”

2. As for a software paper, the authors refer not much about the software. The presented webserver is quite simple. Not much information is given, but only prediction results only. It would be more practical if the method could be applied to whole-genome prediction.

We appreciate the reviewer's feedback regarding the software paper and the desire for more comprehensive information about the presented webserver. We understand the importance of providing detailed information and practical applicability for users.
We expanded the section dedicated to the description of the webserver, providing more information about its functionalities, user interface, and practical usage scenarios. This include details on how users can apply the method to whole-genome prediction, making it more accessible and useful for a wider audience.

We acknowledge the significance of enabling users to apply our method to whole-genome prediction, and we ensure that the manuscript discusses the feasibility and steps involved in such applications. This include guidance on using the webserver for genome-wide predictions. We have updated our network server, incorporating the SSB landscapes of 212 species, and we have introduced Integrated Genome Viewer (IGV) functionality for visualization. In order to align with the research interests of biologists, we have also added features that depict the SSB landscape changes caused by mutations. This allows for a more detailed portrayal of the alterations in the SSB landscape brought about by SNPs, providing research hypotheses for SSB-related studies. We have provided a usage tutorial, and our model is portable, which allows for easy adaptation when new DNA lesion-related data emerges in the future. We also offer a tutorial for retraining the model (https://sxu99.gitbook.io/ssblazer/use-cases/train-a-new-model)

We included information on the availability of resources, such as documentation or tutorials, to assist users in effectively utilizing the webserver and applying the method to their research. These changes could be found in Section SSBlazer web server.
“In this study, we have depicted vast landscapes of SSB sites for diverse species via SSB frequency using SSBlazer, but there are still many unexplored SSB-related applications for this tool. For instance, the SSB landscape within a target gene can be used to evaluate gene stability. To illustrate, we conducted a SNP comparison analysis on Alzheimer’s Disease (AD) patients. We collected pathogenic SNP data from ClinVar [26] and applied SSBlazer to assess how such genomic substitutions might influence the local SSB landscape. Our findings (Figure 4 d) indicate that a particular SNP (rs122356) in ClinVar alters the SSB landscape against the reference genome, suggesting that pathogenic SNPs may promote the occurrence of AD via SSB-related pathways.”

“The current version of SSBlazer trained on the S1 END-seq dataset and the SSiNGLe-ILM dataset while SSBlazer is a portable and well-organized model, which can smoothly transition to the unified dataset and other available SSB detection datasets (e.g., SSB-seq [4], GLOE-seq [5]). We provided a retraining tutorial in https://sxu99.gitbook.io/ssblazer/use-cases/train-a-new-model. This adaptability can substantially improve SSBlazer’s generalization ability, making it a valuable tool in the continued exploration and understanding of the SSBreakome.”

3. As for the proposed method, the novelty is relatively low in algorithms. They applied existing commonly used deep learning models to implement their methods. Moreover, the performance of the proposed models is quite important. As for the predictive performance, it is suggested to compare the proposed methods with more advanced deep learning architectures. Moreover, they should test their proposed methods on the genome-wide annotation?

We appreciate you taking the time to thoroughly review our work and provide thoughtful feedback. You raise a valid point regarding the novelty of the algorithms leveraged in this study. As you noted, we utilized established deep learning architectures rather than introducing entirely new models. However, we also implemented a language model version of SSBlazer (SSBlazer-LM) and demonstrated its ability to achieve comparable performance using a straightforward deep learning framework. While the algorithms themselves may not be highly novel, a key contribution of this work was demonstrating their applicability and efficacy for this particular genomic sequence modeling task and dataset.
“Each model was trained on 1 × NVIDIA A100 GPU with 80 GB memory, and the batch size is 2048. Training time refers to the backpropagation time of the training set of S1 END-seq dataset (imbalance ratio=1, num=231,500), and inference time is the forward propagation time during the testing period (num=22,044). Model AUROC AUPRC MACs Params Training (s) Inference (s) SSBlazer-LM 0.9634 0.7923 43661.70G 110M 422 29 SSBlazer 0.9631 0.7886 332.77G 1.9M 30 8 The growing demand for computing resources in the field of deep learning often limits large-scale downstream applications. This study aims to depict the SSB genomic landscape of various species using a more efficient model. We demonstrate that our model, SSBlazer, achieves performance highly comparable to the BERT-based language model SSBlazer-LM but only requires 0.7% of the computing resources. Our experiment was conducted on 1 × NVIDIA A100 GPU with 80 GB memory using S1 END-seq dataset and determined the batch size as 2048. To qualify the occupied computing resources, MACs (Multiply–Accumulate Operations) was utilized and obtained by thop v.0.0.31 (https://github.com/Lyken17/pytorch-OpCounter). The results indicate that SSBlazer saves 99% MACs and 98% parameters of SSBlazer-LM and maintains a comparable performance (AUROC reduced by 0.0003, AUPRC reduced by 0.0037).

Furthermore, we concur with your suggestion to benchmark against more advanced deep learning techniques in future work. Comparing to state-of-the-art methods would better validate the strengths and limitations of our approach. Expanding the evaluation to additional genomic datasets, as you recommended, would also enhance the generalizability of our methods. In the current study, we did assess performance on GC content prediction, but evaluating on larger-scale genome annotation data could certainly strengthen the conclusions. Thank you again for the insightful feedback. Moving forward, we will prioritize more rigorous algorithmic comparisons and testing across diverse genomic data types to address the limitations you rightfully point out.

4. As for the imbalanced setting, I doubt the authors' settings could simulate the real SSB distributions. They should provide more evidence. Moreover, they should discuss more regarding this issue.

We provided additional evidence and analysis to support the choice of our imbalanced dataset settings. This may include statistical measures, performance metrics, or visual representations to demonstrate the effectiveness of our approach in simulating real SSB distributions. We ensure that the manuscript includes a clear explanation of how the imbalanced dataset settings were designed and justified.

We appreciate the reviewer’s concern about the imbalanced settings in our study and how well they represent real SSB distributions. Given the human genome length of 3,200,000,000 nucleotides, we noted actual SSB ratios varying from 1:103 (as observed in SSiNGLe) to 1:104 (as seen in S1 END-seq). Due to computational resource constraints, we have made every effort to elevate the imbalance ratio to 1000 in an attempt to simulate real-world distributions.We found that too high imbalance ratio will make model miss many of the true SSB sites, so we choose Q=100 for the final model.

Figure R7: (a-d) These profile heatmaps illustrate the effects of varying degrees of imbalanced datasets (Q=1, Q=10, Q=100, Q=1000) on 1,250 confirmed SSB sites, within a 151 bp region surrounding the predicted SSB locations on human genome hg19, chromosome 1. (e) This panel depicts the prediction scores from different models for a specific SSB site region (Human chr1: 871,507 - 871,686), revealing the models' performance under those conditions.

We expanded the discussion section to delve deeper into the implications and limitations of using imbalanced datasets in our study. This include addressing potential biases, challenges, and the generalizability of our findings to real-world scenarios.

5. The writing of the manuscript should be polished further.

We undertook a comprehensive proofreading and language editing process to eliminate grammatical errors, improve sentence structure, and enhance the overall readability of the manuscript. This step involved the assistance of a native English speaker and professional language editing service to ensure the highest quality of written expression.

 
Reference

1. Ji Y, Zhou Z, Liu H, Davuluri RV: DNABERT: pre-trained Bidirectional Encoder Representations from Transformers model for DNA-language in genome. Bioinformatics 2021, 37:2112-2120.
2. Mbatchou J, Barnard L, Backman J, Marcketta A, Kosmicki JA, Ziyatdinov A, Benner C, O’Dushlaine C, Barber M, Boutkov B: Computationally efficient whole-genome regression for quantitative and binary traits. Nature genetics 2021, 53:1097-1103.
3. Hu H, Xiao A, Zhang S, Li Y, Shi X, Jiang T, Zhang L, Zhang L, Zeng J: DeepHINT: understanding HIV-1 integration via deep learning with attention. Bioinformatics 2019, 35:1660-1667.
4. Baranello L, Kouzine F, Wojtowicz D, Cui K, Przytycka TM, Zhao K, Levens D: DNA break mapping reveals topoisomerase II activity genome-wide. International journal of molecular sciences 2014, 15:13111-13122.
5. Sriramachandran AM, Petrosino G, Méndez-Lago M, Schäfer AJ, Batista-Nascimento LS, Zilio N, Ulrich HD: Genome-wide nucleotide-resolution mapping of DNA replication patterns, single-strand breaks, and lesions by GLOE-Seq. Molecular cell 2020, 78:975-985. e977.
6. Zilio N, Ulrich HD: Exploring the SSBreakome: genome‐wide mapping of DNA single‐strand breaks by next‐generation sequencing. The FEBS journal 2021, 288:3948-3961.
7. Benson DA, Cavanaugh M, Clark K, Karsch-Mizrachi I, Lipman DJ, Ostell J, Sayers EW: GenBank. Nucleic acids research 2012, 41:D36-D42.
8. O'Leary NA, Wright MW, Brister JR, Ciufo S, Haddad D, McVeigh R, Rajput B, Robbertse B, Smith-White B, Ako-Adjei D: Reference sequence (RefSeq) database at NCBI: current status, taxonomic expansion, and functional annotation. Nucleic acids research 2016, 44:D733-D745.
9. Wong N, John S, Nussenzweig A, Canela A: END-seq: an unbiased, high-resolution, and genome-wide approach to map DNA double-strand breaks and resection in human cells. Homologous Recombination: Methods and Protocols 2021:9-31.
10. Wu W, Hill SE, Nathan WJ, Paiano J, Callen E, Wang D, Shinoda K, van Wietmarschen N, Colón-Mercado JM, Zong D: Neuronal enhancers are hotspots for DNA single-strand break repair. Nature 2021, 593:440-444.
11. Alipanahi B, Delong A, Weirauch MT, Frey BJ: Predicting the sequence specificities of DNA-and RNA-binding proteins by deep learning. Nature biotechnology 2015, 33:831-838.

**Second round of review**

**Reviewer 1**

All my comments have been addressed.

**Reviewer 2**

The revised version of this manuscript has changed its remit from a research article to a "software" format. I am not familiar with this type of article, so I cannot evaluate whether the standard of this manuscript is adequate for that purpose. The overly long explanations in the rebuttal letter don't help; they rather add to my confusion. My overall comments from a biological point of view have not changed much from the previous version:

- The text is incomprehensible for the non-bioinformatics reader. I therefore doubt that the tool will be used by many biologists.

- The language has not really improved. Even the title cannot stand as it is now: "... model of single-strand break site".

- I take issue with the authors' assumptions about what their tool actually does. Take for once their wording regarding the occurrence of single-stranded breaks in various genomes. They state: "Remarkly, the frequency of SSB is highly variable among different species, ranging from 1,586 in thirteen-lined ground squirrel (Ictidomys tridecemlineatus) to 26,784 in Alaskan stickleback (Gasterosteus aculeatus) and a clear trend along the evolutionary process is observed." How can they make any statement about the "frequency of SSB" from their predictions if they haven't experimentally verified this? First of all, they would have to change "SSB" to "predicted SSB", just to be accurate - and I am quoting only a single instance of this imprecision, which applies to the entire manuscript. More importantly, I think it is misleading to apply their tool to various species and claim that it accurately predicts real breaks when the authors have really no idea at what point or evolutionary distance the assumptions of their model break down. There currently seems to be no way of ensuring the quality of their tool for species for which no experimental SSB maps are available. They don't use any such thing as what would evolutionarily be called an "outgroup" as a control species that clearly does not fall within the range of their prediction.

- The authors acknowledge the value of cooperating with biologists but they have obviously not made any effort to do so.

- Overall, I am not convinced about the usefulness of the authors' tool. I cannot evaluate the correctness of their software, so I am afraid I will not be able to contribute any meaningful comments to the technical aspects.

**Reviewer 3**

All my previous concerns are well addressed. It can be accepted for publication.

**Authors’ response to reviewers**

Reviewer #2: The revised version of this manuscript has changed its remit from a research article to a "software" format. I am not familiar with this type of article, so I cannot evaluate whether the standard of this manuscript is adequate for that purpose. The overly long explanations in the rebuttal letter don't help; they rather add to my confusion. My overall comments from a biological point of view have not changed much from the previous version:

- The text is incomprehensible for the non-bioinformatics reader. I therefore doubt that the tool will be used by many biologists.

Response:
We understand the importance of making our work accessible and relevant to biologists who may not have an extensive background in bioinformatics. So, we have worked collaboratively with biologists to make necessary revisions to ensure that the language and explanations in our manuscript are clear, concise, and understandable to a wider range of readers.

1. Page 5, add a few sentences to emphasize the biological significance of cross-species ability.

The cross-species ability of bioinformatics tools is an indispensable component of modern biological research, offering a powerful means to study organisms that are inherently difficult to access or experimentally manipulate (Ref: 10.1038/s41588-017-0004-9). By leveraging existing genomic data, researchers are allowed to explore the genetic landscapes of inaccessible species and unravel the evolutionary forces shaping their genomes in silico. This ability holds tremendous promise to unlock new biological insights and inform biomedical research across a broad spectrum of species. To this end, we conducted a cross-species benchmarking to evaluate the extensive applications of SSBlazer across diverse species. Since S1 END-seq dataset only covered the Homo sapiens species …

2. Page 7: add a sentence to clearly introduce the capabilities of the integrated gradients method, highlighting its potential applications within the context of this study.

…to determine which nucleotide decides the prediction outcome. The integrated gradients method quantifies each nucleotide’s impact on the model’s prediction by integrating its output gradient, revealing the influence of input variations on the prediction.

3. Page 7: Background on CpG dinucleotides: include a concise explanation of CpG dinucleotides, providing the necessary background associated with SSB.

…CpG dinucleotides are observed to assign significant contribution scores. CpG dinucleotides are specific DNA sequences characterized by the presence of a cytosine (C) followed by a guanine (G) nucleotide, connected by a phosphate group (Ref: 10.1016/j.febslet.2009.04.012). These dinucleotides serve as pivotal elements in epigenetic regulation, exerting influence over the stability of the genome by modulating DNA repair processes (Ref: 10.1016/j.mrrev.2017.10.001). We observed an enhanced frequency of CpG dinucleotides in an SSB site context, which is consistent with the genome-wide landscape that SSBs are intended to locate at or near CpG dinucleotides [18].

4. Page 10: add additional sentences to illustrate the potential relationship between single nucleotide polymorphisms (SNPs) and single-strand breaks (SSBs).

…but there are still many unexplored SSB-related applications for this tool. One intriguing possibility is investigating changes in the SSB landscape within a genomic region can provide valuable insights into the mechanisms of how the host gene or nearby SNP contributes to disease progression. To illustrate, we conducted a SNP comparison analysis on Alzheimer’s Disease (AD) patients. Emerging evidence suggests that genetic factors, including single nucleotide polymorphisms (SNPs), play a critical role in AD susceptibility (Ref: 10.1016/S0168-9525(01)02342-3; 10.1038/tp.2012.45). However, specific mechanisms of SNPs in AD development are still being understudied. In this study, we collected pathogenic SNP data from ClinVar…

We also recognize that the usability of any tool depends not only on its technical capabilities but also on its accessibility and user-friendliness. Therefore, we are committed to update our user interface to facilitate its utilization by biologists.

We updated Guide page on the webserver:
https://proj.cse.cuhk.edu.hk/aihlab/ssblazer/#/guide/

The step-by-step user manual is available on tutorial page:
https://sxu99.gitbook.io/ssblazer/web-server-manual/break-sites-prediction

- The language has not really improved. Even the title cannot stand as it is now: "... model of single-strand break site".

Response:
We have undertaken a comprehensive review of our manuscript with a focus on simplifying the language for better understanding while maintaining scientific accuracy. We have also refined the title to better reflect the content:

SSBlazer: A genome-wide nucleotide-resolution model for predicting single-strand break sites.

- I take issue with the authors' assumptions about what their tool actually does. Take for once their wording regarding the occurrence of single-stranded breaks in various genomes. They state: "Remarkly, the frequency of SSB is highly variable among different species, ranging from 1,586 in thirteen-lined ground squirrel (Ictidomys tridecemlineatus) to 26,784 in Alaskan stickleback (Gasterosteus aculeatus) and a clear trend along the evolutionary process is observed." How can they make any statement about the "frequency of SSB" from their predictions if they haven't experimentally verified this? First of all, they would have to change "SSB" to "predicted SSB", just to be accurate - and I am quoting only a single instance of this imprecision, which applies to the entire manuscript. More importantly, I think it is misleading to apply their tool to various species and claim that it accurately predicts real breaks when the authors have really no idea at what point or evolutionary distance the assumptions of their model break down. There currently seems to be no way of ensuring the quality of their tool for species for which no experimental SSB maps are available. They don't use any such thing as what would evolutionarily be called an "outgroup" as a control species that clearly does not fall within the range of their prediction.

Response:
We fully understand the reviewer’s concern about the potential breakdown of our model at a certain evolutionary distance and the lack of outgroup control. Introducing an outgroup control species can indeed provide additional insights into the performance of our tool and help assess the robustness of our predictions. However, it is important to note that the choice of an appropriate outgroup control species can be challenging. The selection of an outgroup should balance phylogenetic distance to exhibit distinct SSB patterns while still sharing sufficient evolutionary conservation. If the outgroup is too distant, it may introduce confounding factors that affect the comparison, such as differences in repair mechanisms. Furthermore, the availability of high-quality genomic data for the outgroup species may be limited or absent.

Nevertheless, our tool has consistently shown promising results in human with the available ground truth SSB data as well as the applicability for other species. The metrics we used provide quantitative measures of accuracy, precision, sensitivity, and specificity, further supporting the high performance of our tool. Admittedly, as the reviewer points out, we cannot guarantee the performance of our tool in species lacking experimental SSB maps. Given the current constraints, we would like to emphasize that our tool should be considered a valuable resource for generating hypotheses and guiding subsequent experimental validation, rather than providing definitive conclusions about the occurrence of real breaks in all species.

In addition, our study has several limitations. A primary limitation is the current reliance on human data for validation, utilizing available ground truth datasets. While the complexity of the human genome provides a strong foundation for evaluating prediction performance, we recognize the need for broader validation across different species. Additionally, the absence of an outgroup control species is an important consideration. Despite these limitations, we believe our current findings still contribute to the understanding of SSB patterns and the potential applications of our tool.

- The authors acknowledge the value of cooperating with biologists but they have obviously not made any effort to do so.

Response:
We have actively collaborated with biologists to ensure biological relevance and accuracy during the tool evaluation and results interpretation processes. We would like to clarify that our study focused on developing and evaluating a computational tool for predicting SSBs based on available genomic data and ground truth datasets. While experimental validation is indeed valuable and important in biological research, it is beyond the scope of this study. By clearly delineating the objectives and limitations of our research, we aim to contribute to the field and provide a foundation for guiding future experimental studies in this area.

- Overall, I am not convinced about the usefulness of the authors' tool. I cannot evaluate the correctness of their software, so I am afraid I will not be able to contribute any meaningful comments to the technical aspects.

Response:
Thank you for your diligent review of our manuscript and for your candid feedback. As a biologist, your perspective is particularly valuable to us in our endeavor to make our tool widely accessible and user-friendly, not only for computational experts but also for biologists who may not be as familiar with computational tools. To this end, we have carefully assembled detailed instructions on our web server (Ref: https://proj.cse.cuhk.edu.hk/aihlab/ssblazer/#/guide/) and developed a comprehensive GitBook tutorial (Ref: https://sxu99.gitbook.io/ssblazer/). These resources are tailored to guide all users, regardless of their computational background, with clear, step-by-step instructions, use-case examples, and visual aids that elucidate the various functionalities of our software.
